# Post-exposure prophylaxis against SARS-CoV-2 in close contacts of confirmed COVID-19 cases (CORIPREV): study protocol for a cluster-randomized trial

**DOI:** 10.1186/s13063-021-05134-7

**Published:** 2021-03-22

**Authors:** Darrell H. S. Tan, Adrienne K. Chan, Peter Jüni, George Tomlinson, Nick Daneman, Sharon Walmsley, Matthew Muller, Rob Fowler, Srinivas Murthy, Natasha Press, Curtis Cooper, Todd Lee, Tony Mazzulli, Allison McGeer

**Affiliations:** 1 Division of Infectious Diseases, St. Michael’s Hospital, Toronto, Canada; 2 MAP Centre for Urban Health Solutions, St. Michael’s Hospital, Toronto, Canada; 3 Department of Medicine, University of Toronto, Toronto, Canada; 4 Division of Infectious Diseases, University Health Network, Toronto, Canada; 5 Institute of Health Policy, Management and Evaluation, Dalla Lana School of Public Health, University of Toronto, Toronto, Canada; 6 Division of Infectious Diseases, Sunnybrook Hospital, Toronto, Canada; 7 Applied Health Research Centre, St. Michael’s Hospital, Toronto, Canada; 8 Department of Medicine, University Health Network, Toronto, Canada; 9 Department of Medicine, Sunnybrook Hospital, Toronto, Canada; 10 Department of Pediatrics, University of British Columbia, Vancouver, Canada; 11 Division of Infectious Diseases, St. Paul’s Hospital, Vancouver, Canada; 12 Division of Infectious Diseases, Ottawa Hospital Research Institute, Ottawa, Canada; 13 Division of Infectious Diseases, McGill University Health Centre, Montreal, Canada; 14 Department of Microbiology, Mount Sinai Hospital/University Health Network, Toronto, Canada; 15 Department of Laboratory Medicine and Pathobiology, University of Toronto, Toronto, Canada

**Keywords:** COVID-19, Randomized controlled trial, Protocol, Cluster randomization, Post-exposure prophylaxis, Chemoprophylaxis, Lopinavir/ritonavir

## Abstract

**Background:**

Post-exposure prophylaxis (PEP) is a well-established strategy for the prevention of infectious diseases, in which recently exposed people take a short course of medication to prevent infection. The primary objective of the COVID-19 Ring-based Prevention Trial with lopinavir/ritonavir (CORIPREV-LR) is to evaluate the efficacy of a 14-day course of oral lopinavir/ritonavir as PEP against COVID-19 among individuals with a high-risk exposure to a confirmed case.

**Methods:**

This is an open-label, multicenter, 1:1 cluster-randomized trial of LPV/r 800/200 mg twice daily for 14 days (intervention arm) versus no intervention (control arm), using an adaptive approach to sample size calculation. Participants will be individuals aged > 6 months with a high-risk exposure to a confirmed COVID-19 case within the past 7 days. A combination of remote and in-person study visits at days 1, 7, 14, 35, and 90 includes comprehensive epidemiological, clinical, microbiologic, and serologic sampling. The primary outcome is microbiologically confirmed COVID-19 infection within 14 days after exposure, defined as a positive respiratory tract specimen for SARS-CoV-2 by polymerase chain reaction. Secondary outcomes include safety, symptomatic COVID-19, seropositivity, hospitalization, respiratory failure requiring ventilator support, mortality, psychological impact, and health-related quality of life. Additional analyses will examine the impact of LPV/r on these outcomes in the subset of participants who test positive for SARS-CoV-2 at baseline. To detect a relative risk reduction of 40% with 80% power at *α* = 0.05, assuming the secondary attack rate in ring members (*p*_0_) = 15%, 5 contacts per case and intra-class correlation coefficient (ICC) = 0.05, we require 110 clusters per arm, or 220 clusters overall and approximately 1220 enrollees after accounting for 10% loss-to-follow-up. We will modify the sample size target after 60 clusters, based on preliminary estimates of *p*_0_, ICC, and cluster size and consider switching to an alternative drug after interim analyses and as new data emerges. The primary analysis will be a generalized linear mixed model with logit link to estimate the effect of LPV/r on the probability of infection. Participants who test positive at baseline will be excluded from the primary analysis but will be maintained for additional analyses to examine the impact of LPV/r on early treatment.

**Discussion:**

Harnessing safe, existing drugs such as LPV/r as PEP could provide an important tool for control of the COVID-19 pandemic. Novel aspects of our design include the ring-based prevention approach, and the incorporation of remote strategies for conducting study visits and biospecimen collection.

**Trial registration:**

This trial was registered at www.ClinicalTrials.gov (NCT04321174) on March 25, 2020.

**Supplementary Information:**

The online version contains supplementary material available at 10.1186/s13063-021-05134-7.

## Administrative information

**Table:** 

Title {1}	CORIPREV-LR: Study protocol for a cluster-randomized trial of post-exposure prophylaxis against SARS-CoV-2 in close contacts of confirmed COVID-19 cases
Trial registration {2a and 2b}.	This trial is registered at www.clinicaltrials.gov (NCT04321174).
Protocol version {3}	Version 1.5, 9 June 2020
Funding {4}	Grant funding: Canadian Institutes of Health Research St. Michael’s Hospital Foundation In-kind support: Abbvie CIHR Centre for REACH 3.0
Author details {5a}	Darrell H. S. Tan,^1–5^ Adrienne K. Chan,^3, 5, 6^ Peter Jüni, ^3, 5, 7^ George Tomlinson,^8^ Nick Daneman,^3, 6^ Sharon Walmsley,^3–5^ Matthew Muller,^1, 3^ Rob Fowler,^3, 5, 9^ Srinivas Murthy,^10^ Natasha Press,^11^ Curtis Cooper,^12^ Todd Lee,^13^ Tony Mazzulli,^14, 15^ Allison McGeer^3, 14^ 1. Division of Infectious Diseases, St. Michael’s Hospital 2. MAP Centre for Urban Health Solutions, St. Michael’s Hospital 3. Department of Medicine, University of Toronto 4. Division of Infectious Diseases, University Health Network 5. Institute of Health Policy, Management and Evaluation, Dalla Lana School of Public Health, University of Toronto, Canada 6. Division of Infectious Diseases, Sunnybrook Hospital 7. Applied Health Research Centre, St. Michael’s Hospital 8. Department of Medicine, University Health Network 9. Department of Medicine, Sunnybrook Hospital 10. Department of Pediatrics, University of British Columbia 11. Division of Infectious Diseases, St. Paul’s Hospital 12. Division of Infectious Diseases, Ottawa Hospital Research Institute 13. Division of Infectious Diseases, McGill University Health Centre 14. Department of Microbiology, Mount Sinai Hospital/University Health Network 15. Department of Laboratory Medicine and Pathobiology, University of Toronto
Name and contact information for the trial sponsor {5b}	Sponsor-Investigator: Dr. Darrell H. S. Tan darrell.tan@gmail.com (416) 864-5568
Role of sponsor {5c}	The study funders, including those providing in-kind support for the trial, have no role in the study design; collection, management, analysis, and interpretation of data; writing of the report; and the decision to submit the report for publication. The sponsor-investigator for this trial is also the Principal Investigator, and as such is primarily responsible for the study design; collection, management, analysis, and interpretation of data; writing of the report; and the decision to submit the report for publication. The Steering Committee will have ultimate authority over all of these activities.

## Introduction

### Background and rationale {6a}

The lack of population immunity to SARS-CoV-2, potential for pauci-symptomatic and asymptomatic transmission, basic reproductive number of roughly 2.5 [1], case fatality estimates of up to 7.2% [2], and lack of evidence-based therapies mean that the prevention of new COVID-19 cases is critical to controlling the pandemic. Encouragingly, highly effective preventive vaccines have been developed and are already being distributed in settings worldwide [3, 4]. However, the high transmissibility of the pathogen and the many implementation challenges in achieving high vaccine coverage mean that combination approaches to COVID-19 prevention will remain important in the future. Post-exposure prophylaxis (PEP) is a well-established strategy for the prevention of infectious diseases such as HIV, in which recently exposed people take a short course of medication to prevent infection. COVID-19 PEP has been identified early as a research priority by the World Health Organization (WHO) [5].

Several lines of evidence suggest that the antiretroviral lopinavir/ritonavir (LPV/r, marketed in Canada as Kaletra™) is one drug that may have meaningful antiviral activity against coronaviruses such as SARS-CoV-2, which causes COVID-19. Molecular data show that LPV/r has activity against the SARS-CoV protease M^pro^ [6], while in vitro and animal data show that it has activity against the closely related Middle East respiratory syndrome coronavirus (MERS-CoV) [7]. A small observational study in South Korea suggested that LPV/r PEP decreased the risk of infection in healthcare workers exposed to MERS-CoV [8].

An initial clinical trial in China found no definite treatment benefit of LPV/r in hospitalized COVID-19 patients [9] but was underpowered for the primary endpoint of mortality, and initiation of LPV/r was late in the course of COVID-19 disease. There was also a potential for imbalance in baseline characteristics (higher viral load, later presentation, more severe illness in the LPV/r arm) to influence the outcomes. Secondary endpoints hinted at potential benefit (numerically lower mortality, shorter ICU/hospital stay) [9]. As noted by many observers [10–14], the results underscore the need for more LPV/r trials. While subsequent results from the SOLIDARITY and RECOVERY trials showed no impact of treatment with LPV/r on mortality in hospitalized COVID-19 patients [15, 16], data are lacking on the impact of this drug when used for prophylaxis or pre-emptive therapy.

The safety of LPV/r is already well-established, since it has been widely used in HIV treatment and PEP for over 20 years [17–19]. While it can cause temporary gastrointestinal side effects [20–23], discontinuation due to side effects was uncommon in HIV PEP trials [24–26]. Drug interactions are well understood [27], although the large number of potential interacting agents can pose clinical challenges. LPV/r is safe in pregnancy, liver disease and at all levels of renal function including dialysis and is available in liquid form to facilitate use in children and adults who cannot take pills/tablets [27]. It is also available worldwide, including in resource-limited settings. Thus, the feasibility of LPV/r as COVID-19 PEP is high if shown to be effective, and it is critical to develop evidence regarding its use.

This trial will examine the efficacy and safety of a 14-day course of LPV/r as PEP in close contacts of individuals with confirmed SARS-CoV-2 infection. All items in the WHO trial registry data set can be found in the protocol (Appendix A).

### Objectives {7}

The primary objective of this trial is to evaluate the efficacy of a 14-day course of oral LPV/r as PEP against microbiologically confirmed COVID-19 among individuals with a significant unprotected exposure to a confirmed case.

Secondary objectives are

1. To compare the following secondary outcomes between study arm:

- Adverse events as defined by the DAIDS Table for Grading the Severity of Adverse Events [28];
- Symptomatic COVID-19 disease;
- Seroconversion;
- Hospitalization;
- Respiratory failure requiring (i) non-invasive or (ii) invasive ventilation
- Mortality;
- Short-term psychological distress associated with COVID-19 exposure;
- Long-term psychological distress associated with COVID-19 exposure; and
- Health-related quality of life.

2. To compare clinical outcomes associated with early LPV/r treatment among participants with baseline COVID-19 infection.
3. To characterize key transmission-related epidemiologic parameters among exposed contacts in a Canadian context, including exposure histories, cluster size, secondary attack rate (p_0_, incidence proportion), time to first viral shedding and burden, risk factors for transmission, and correlates of symptomatic disease.

### Trial design {8}

The COVID-19 Ring-based Prevention trial with Lopinavir/ritonavir (CORIPREV-LR) is an open-label, cluster-randomized trial of lopinavir/ritonavir versus no intervention for preventing microbiologically confirmed SARS-CoV-2 infection in high-risk close contacts of confirmed COVID-19 cases. The CORIPREV trial will use an adaptive approach to the selection of study drug, with consideration of switching to an alternative promising agent after interim analyses are completed and/or considering external data.

The trial will use a ring-based approach to delivering these preventive strategies, adapting a novel cluster trial design recently used in the 2013–16 West African Ebola epidemic in the *Ebola ça suffit*! trial [29, 30]. The approach is to define a ‘ring’ of exposed contacts around COVID-19 cases, and to randomize rings (i.e., clusters) to study drug or control (no study drug) [31]. This design maximizes statistical power by recruiting those at highest risk. It also has the potential to create a buffer of protected people around cases, such that if the drug works for prevention, the trial itself may limit transmission. Further, some COVID-19 cases may lead to “super-spreading” events [32, 33]; this variability is taken into account by defining index cases, rather than exposed contacts, as the unit of randomization. Ring vaccination has been a key part of the successful eradication of other highly transmissible infectious diseases such as smallpox [34].

## Methods: participants, interventions, and outcomes

### Study setting {9}

Initially, the trial will enroll community-based participants in Toronto, Ottawa, Montreal, and Vancouver, Canada. Study staff will be based at academic hospital sites but will conduct the majority of study visits remotely (via video-link or telephone), as per the schedule of events provided below. Additional sites may be opened in other cities depending on the degree of epidemic activity.

### Eligibility criteria {10}

Inclusion criteria for the trial are as follows:

1. Within the past 1–7 days, high-risk close contact with a confirmed COVID-19 case. If the index case was symptomatic, this contact must have occurred during their symptomatic period, including 1 day before symptom onset. If the index case was asymptomatic, this contact must have occurred within 14 days of the index case’s first positive SARS-CoV-2 test. High-risk close contact is defined as any of the following exposures without the consistent appropriate use of recommended personal protective equipment (e.g., face mask):
  - Provided direct care for the index case;
  - Had close direct physical contact with the index case;
  - Lived with the index case;
  - Had close indoor contact (within 2 m), with or without direct physical contact, for at least 1 h; and
  - Had direct contact with infectious body fluids, including oral secretions, respiratory secretions, or stool.
    This definition is in accordance with the definition of a high-risk close contact for COVID-19 set out by the Public Health Agency of Canada [35].

2. Successfully contacted by the study team within 24 h of study team notification of the relevant index COVID-19 case. This time window is necessary because the efficacy of PEP may be dependent on the timing of its initiation, and because randomization of a ring cannot be delayed while awaiting response from contacts that cannot be rapidly reached.
3. Age ≥ 6 months, since the safety and pharmacokinetic profiles of LPV/r in pediatric patients below the age of 6 months have not been established.
4. Ability to communicate with study staff in the language(s) of the study site

Exclusion criteria are as follows:

1. Known hypersensitivity/allergy to lopinavir or ritonavir.
2. Current use of LPV/r for the treatment or prevention of HIV infection.
3. Receipt of LPV/r in the context of this trial or any other trial of COVID-19 PEP within 2 days or less prior to the last known contact with the index COVID-19 case. The 2 day time window is intended to ensure that exposure would not have occurred in the presence of clinically relevant drug levels (five times the elimination half-life of LPV/r, which is estimated at 4–6 h with prolonged use).
4. Currently breastfeeding an infant, due to potential for serious adverse reactions in nursing infants exposed to variable levels of LPV/r in breastmilk.
5. Concomitant medications with prohibited drug interactions with LPV/r that cannot be temporarily suspended/replaced (see full study protocol in Appendix A) [27].
6. Already known to be infected with SARS-CoV-2.
7. Receipt of any doses of any locally licensed COVID-19 vaccine.

Enrolled participants whose baseline samples are positive for COVID-19 will continue all study procedures (including study drug) but be excluded from the primary analysis and included instead in the secondary analysis regarding LPV/r as early treatment.

### Who will take informed consent? {26a}

Informed consent will be sought from potential participants by trained research coordinators at study sites. The informed consent form can be accessed online at https://optionslab.ca/projects/coriprev/. The Aid To Capacity Evaluation (ACE) tool [36] will be used to assess capacity in adults as needed, or in children aged 10 and over. Children aged 9 and under will be presumed incapable and informed consent to participate should be sought through their substitute decision-maker.

### Additional consent provisions for collection and use of participant data and biological specimens {26b}

As part of the informed consent process, participants are informed that the study protocol includes the collection of serum and plasma specimens for long-term storage. Samples will be stored in a biobank for potential future studies related to respiratory pathogens and inflammatory biomarkers.

## Interventions

### Explanation for the choice of comparators {6b}

The control condition is no intervention, which is ethically justified because no agent has definitive proof of efficacy in preventing COVID-19 (see further comments regarding practical and scientific considerations provided below under “blinding”).

### Intervention description {11a}

The intervention is a 14-day course of LPV/r 400/100 mg (administered as two 200/50 mg tablets) orally twice daily, to be initiated as soon as possible and no later than 8 days after the latest exposure to the index case. Individuals 6 months of age and older who are unable to swallow tablets will be administered the liquid formulation of LPV/r, at equivalent weight-based dosing according to the product monograph [37]. The 14-day duration is based on COVID-19’s estimated incubation period of 6.4 days (95% credible interval = 5.6–7.7) [38], with a maximum expected incubation period similar to SARS/MERS at 14 days [39, 40].

This dosing regimen was selected based on extensive clinical experience in the setting of HIV treatment and prevention. Of note, in the setting of HIV treatment, an alternative dosing strategy of 800/200 mg once daily has been evaluated and shown to be non-inferior to twice daily dosing for that indication [41]. However, because the pharmacodynamics for LPV/r’s potential antiviral activity against SARS-CoV-2 are not fully understood, this trial will recommend twice daily dosing.

### Criteria for discontinuing or modifying allocated interventions {11b}

There are no protocol-defined criteria for discontinuing or modifying the study interventions. However, a special circumstance may arise if one individual is identified as part of more than one ring, either sequentially or simultaneously. The approach taken in these situations will be based on the temporal sequence in which study staff make contact with the individual (not the timing of the person’s exposure to the relevant index cases).

If notification of this situation to the participant occurs sequentially, and the participant’s initial ring had already been randomized to the control situation (no intervention), then for ethical reasons, the participant should be given the choice of whether to re-enroll in the second ring, since this re-enrollment may provide the opportunity to be randomized to the study drug. The decision regarding re-enrollment in the second ring must be made prior to randomization of that second ring; once randomization has occurred the participant cannot switch their ring affiliation. If the participant is already taking study drug at the time they are notified about membership in a second ring, then they are not eligible to re-enroll in the second ring. If the participant is not currently taking study drug at the time of notification, they may be eligible to re-enroll if all inclusion and exclusion criteria are met.

If a participant is simultaneously notified about membership in two or more rings, the participant should be offered the choice of which ring they wish to be affiliated with. This decision must be made prior to randomization of either ring. It is anticipated that such decisions may be based purely on convenience considerations (e.g., a participant may wish to be affiliated with the same ring as other family members).

### Strategies to improve adherence to interventions {11c}

Participants in the active arm of the trial will receive a few doses of dimenhydrinate 50 mg (for management of nausea) and loperamide 2 mg (for diarrhea) in their study kits, in order to help manage potential gastrointestinal side effects of the study drug.

Of note, the study team considered a loading dose of LPV/r at baseline (400/100 mg four times on day 1, followed by twice daily to complete 14 days total) in order to optimize the pharmacokinetics of this drug against SARS-CoV-2. However, this strategy was not selected due to concern that it may be associated with an increased risk of side effects that could compromise further adherence with the study drug.

Adherence will be monitored in two ways. First, participants in the active arm will be asked to complete a daily electronic symptom diary which asks whether each dose was early, on time (within 3 h), late, or forgotten/missed. Second, a random ~ 30% subset of participants in the active arm will have a self-collected dried blood spot collected at day 14 for testing of plasma lopinavir levels as a biomarker of adherence and to assess for a pharmacokinetic correlate of protection. Because these levels can be affected by hematocrit [42, 43], this subset of participants will also undergo a complete blood count at the day 35 in-person visit, together with the routine blood draw for serology.

### Relevant concomitant care permitted or prohibited during the trial {11d}

Use of LPV/r together with medications cleared through cytochrome P450 3A (CYP 3A) can lead to elevated plasma concentrations of the concomitant medication. LPV/r also induces glucuronidation which may affect the exposure of some drugs. Medications that inhibit or induce CYP3A may increase or decrease serum concentrations of LPV/r, respectively. These drug-drug interactions may lead to adverse reactions, loss of therapeutic effect of the concomitant medication, and/or loss of antiviral activity of LPV/r.

Site investigators must therefore review concomitant medications at the time of enrollment. A full list of contraindicated medications appears in the study protocol in Appendix A [37], and the use of any of these products that cannot be substituted during the study dosing period is an exclusion criterion for the study. For other medications and/or supplements that are not on the list of contraindicated medications, site investigators must assess the risk of drug-drug interactions, and adjust medication doses accordingly. Study pharmacists with extensive experience with LPV/r use will provide advice to investigators regarding the management of drug-drug interactions as needed, via email or telephone consultation.

Co-enrollment into other regulated clinical trials of COVID-19 prevention is permitted, but details of the co-intervention must be documented under the concomitant medication section or other suitable part of the database.

### Provisions for post-trial care {30}

This study will provide access to 14 days of the study drug for those randomized to the intervention arm only. Because a secondary objective of the trial is to examine the impact of LPV/r on clinical outcomes in those who test positive for SARS-CoV-2, the full course of study drug should be continued regardless of the results of COVID-19 testing.

In the event that a participant tests positive for SARS-CoV-2 during or after the follow-up period, efforts will be made to link the individual to ongoing clinical trials of COVID-19 treatment. However, no guarantee of access to investigational product can be made. At the time of writing, there are no known effective anti-viral treatments for established COVID-19 infection, although dexamethasone has been shown to improve 28-day mortality in hospitalized patients requiring oxygen or invasive mechanical ventilation [44].

### Outcomes {12}

The primary outcome is microbiologically confirmed SARS-CoV-2 infection, i.e., the proportion of participants with detection of SARS-CoV-2 RNA by polymerase chain reaction (PCR) in a respiratory specimen (oropharyngeal/nasal swab, nasopharyngeal swab, sputum specimen, saliva specimen, oral swab, endotracheal aspirate, bronchoalveolar lavage specimen) by day 14 of the study. This is the most relevant outcome for this PEP trial, since it represents incident SARS-CoV-2 infection. Specimens will be collected on all participants at baseline, day 7, day 14, and within 24 h of new symptoms, as detailed further below.

Secondary outcomes are:

- Adverse events: the proportion of participants exhibiting adverse events of any grade as defined using the DAIDS Table for Grading the Severity of Adverse Events, at 7, 14, 35, and 90 days [28].
- Symptomatic microbiologically confirmed SARS-CoV-2 infection: proportion of participants with fever, cough, or other respiratory/systemic symptoms (including but not limited to fatigue, myalgias, arthralgias, shortness of breath, sore throat, headache, chills, coryza, nausea, vomiting, diarrhea), combined with microbiologic confirmation of SARS-CoV-2 infection by PCR, by day 14.
- Seropositivity: proportion of participants with reactive serology at day 35.
- Days of hospitalization attributable to COVID-19 disease: The median number of days (or partial days) spent admitted to an acute care hospital will be tabulated both at day 35 and day 90.
- Respiratory failure requiring ventilatory support attributable to COVID-19 disease: The median number of days (or partial days) requiring (i) non-invasive or (ii) invasive ventilation will be tabulated both at day 35 and day 90.
- Mortality (proportion of participants who die) attributable to COVID-19 disease and all-cause mortality will be tabulated at 35 and 90 days.
- Short-term psychological impact of exposure to COVID-19 will be measured at day 14 using the K10, a validated measure of non-specific psychological distress, with results aggregated as the proportion with a score of ≥ 16 [45, 46].
- Long-term psychological impact of exposure to COVID-19 will be measured at day 90 using the Impact of Event Scale, a validated measure of traumatic stress response, with results aggregated as the proportion with a score of ≥ 26 [47, 48].
- Health-related quality of life will be measured using the EQ-5D-5L (EuroQol-5D) (https://euroqol.org/eq-5d-instruments/eq-5d-5l-about/). The EQ-5D consists of two pages: the EQ-5D descriptive system and the EQ visual analog scale (EQ VAS). The descriptive system comprises five dimensions: mobility, self-care, usual activities, pain/discomfort and anxiety/depression. The tool will be administered to participants at 1, 14, 35 and 90 days; the primary interest is in the median index value at 14 days.

### Participant timeline {13}

Potential participants are screened for study eligibility by telephone ± video link; consenting individuals undergo a remote (via video-link or, if not feasible, by telephone) baseline visit (day 1) within 24 h. Subsequent remote visits occur on days 7, 14, and 90; a single in-person visit is requested at day 35 to draw blood. The full schedule of events is shown in Table 1.

**Table 1:** Schedule of events for the CORIPREV-LR trial

Visit	Screening (day 0)	Baseline (day 1)	Day 7, ± 2 days	Day 14, ± 2 days	Day 35, ± 4 days	Day 90, ± 2 days
**Visit format**	**Remote**	**Remote**	**Remote**	**Remote**	**In person**	**Remote**
Eligibility assessment	X					
Informed consent/assent	X					
Cluster randomization	X^a^					
Dispensation of study drug (LPV/r arm)		X				
Interview by study staff		X	X	X	X	X
Concomitant medication assessment		X	X	X	X	X
Adverse event assessment			X	X	X	X
Adherence diary (LPV/r arm)		Daily on d1–14				
Symptom diary		Daily on d2–15, then d21, d28, d35				
Temperature diary		Daily on d1–14				
Visit-specific questionnaire^b^		X		X	X	X
HIV self-test^c^		X				
Self-collected saliva sample		X	X	X	X	
Self-collected oropharyngeal/nasal swab^d^		X	X	X		
Self-collected dried blood spot		X^e^		X^f^		
Blood for SARS-CoV-2 serology		X^g^			X	
Stored blood samples					X	
Blood for CBC					X^f^	

^a^Randomization of the cluster occurs once all exposed contacts of an index COVID-19 case have provided their decision about study participation, or could not be contacted within 24 h of the study team becoming aware of the index case; once randomization has been completed then site coordinators should contact individual participants to conduct the baseline activities

^b^Specific components of each visit-specific questionnaire are listed in sections 13.3–13.7 below. Questionnaires can be completed on paper or via interview if internet access is unavailable. Guardians may complete questionnaires together with minors if preferred/not feasible by the minor

^c^ The HIV Self-test should only be done in participants over the age of 18 months; those under this age and those unable to perform the self-test for any reason (e.g., extreme needle phobia, limited manual dexterity) should undergo standard HIV testing at day 35 (HIV RNA test in those aged < 18 months; 4th generation antibody/antigen test for all others)

^d^An additional swab is to be collected within 24 h if symptoms develop before day 14. Two respiratory specimens (swabs, saliva, etc.) are to be taken at day 14 timepoint, unless an extra swab was already taken due to the development of symptoms in which case only one is collected

*NB:* other respiratory specimen(s) may be collected depending on availability of supplies

^e^The dried blood spot at baseline is to be performed in participants in the active arm only (since these participants are already collecting a drop of blood for the HIV self-test), for SARS-CoV-2 serology

^f^The dried blood spot at day 14 is to be performed in a random subsample of participants in the active arm only, for lopinavir levels. These participants are the only ones that require a CBC at day 35 since the hematocrit is required for interpretation of DBS lopinavir levels [42, 43]

^g^Baseline serology is to be collected at baseline only if the participant is institutionalized

### Sample size {14}

Our target sample size is 244 rings, assuming an average ring size of 5 individuals (i.e., a total of *n* = 1220 participants), an intra-class correlation coefficient (ICC) of 0.05 [31], a secondary attack rate (proportion of those exposed who get infected, p_0_) of 15%, and inflation for 10% loss-to-follow-up. This sample size will allow us to detect whether the study drug decreases the relative risk of COVID-19 by at least 40% (effect size estimated in the MERS-CoV LPV/r PEP study [8]), with 80% power and alpha = 0.05.

The number of contacts per case may vary considerably but was estimated at 5 based on published data for SARS in Beijing (mean = 3.8) [49]. We considered ICC values of 0.02 and 0.05 [31]. The estimated secondary attack rate of 15% was originally estimated based on transmission rates for SARS [49–52] and influenza [53–56] and is corroborated by recent studies on COVID-19. Among 19 household contacts of the first 10 patients with travel-related COVID-19 in the United States, *p*_0_ = 10.5% (95%CI = 2.9%–31.4%) [57]. In Shenzhen, the secondary attack rate among 686 household contacts was 11.2% (95%CI = 9.1–13.8) [58]. In Guangzhou, it was 19.3% (95%CI = 15.5–23.9%) among household contacts sharing a residential address with a case [59]. Investigators in Wuhan found secondary transmission in 64/392 household contacts (16.3%) [60]. Illness compatible with COVID-19 developed in 58/407 (14.8%) of placebo arm participants in a recent trial of hydroxychloroquine as COVID-19 PEP [61].

The parameters *p*_0_, ICC and cluster size are important drivers of statistical power [62, 63], but are still being elucidated for COVID-19 and may vary according to context. We will thus apply an adaptive trial design [63] to revise our sample size calculations based on day 14 data from the first 60 clusters.

Another interim analysis will be done when day 14 data for 50% of the target sample have accrued; our independent data safety monitoring committee will advise on trial continuation/switching to a different agent, taking external data into account, and through additional confidential consultation with the WHO Core DSMC on COVID-19 prophylaxis, with which we are linked [5].

### Recruitment {15}

Enrollment of exposed persons will typically occur by first identifying new lab-confirmed COVID-19 cases, and then conducting contact tracing to define a ring of exposed contacts around those index cases. The study team will actively seek out such new cases by building referral pathways from COVID-19 Assessment Centres, Emergency Departments, inpatient units, occupational health departments, and public health authorities in the vicinity of each study site. Once the list of exposed contacts for each case is identified, the study team will make multiple attempts to contact them for up to 24 h, in order to obtain their decision about trial participation.

In addition, the team will post advertisements on social media seeking both individuals who have been newly diagnosed, as well as people who have recently been exposed to a confirmed case. As the epidemic evolves, the study team will consider expansion of the trial to other study sites in jurisdictions with increasing numbers of cases.

## Assignment of interventions: allocation

### Sequence generation {16a}

Rings will be randomized to the intervention or control conditions in a 1:1 ratio in permuted blocks of variable size and stratified by study site, according to a computer-generated sequence of random numbers. Block sizes will not be revealed to study staff.

### Concealment mechanism {16b}

Randomization will be implemented using a secure interactive web-based response system (Research Electronic Data Capture, REDCap). To ensure allocation concealment, the randomization sequence is fully concealed, and treatment assignment will not be revealed until the moment of randomization. Study personnel will then notify participants about their assigned study arm at the time of the baseline visit.

### Implementation {16c}

The allocation sequence was generated by a statistician not otherwise involved in the trial, using a computer-generated sequence of random numbers. That statistician is the only person who will have access to the study codes. When interim analyses are conducted, only the codes related to the participants to be included in the interim analysis will be released to the biostatistician from the Data Safety and Monitoring Committee.

Study coordinators will use the web-based system to randomize rings of participants. An automated audit trail will record the time, date, allocation, and participant identification numbers. Each ring will only be randomized once all members of a ring either: give preliminary consent, decline or are classified as not contactable within the 24 h window for obtaining consent. It is essential to secure preliminary consent of the individual participants in a ring before randomization of the cluster in order to avoid selection bias, since this study is open-label.

## Assignment of interventions: blinding

### Who will be blinded {17a}

This trial is open-label at the level of the participant, study coordinator, and investigator due to the impracticality of securing a supply of placebo. However the primary outcome is an objective biological event (microbiologic evidence of virus by RT-PCR), and lab technologists and statisticians will be blinded through the use of a randomly generated secondary participant identification number that is unlinked to cluster.

### Procedure for unblinding if needed {17b}

Unblinding is not applicable to this open-label trial.

## Data collection and management

### Plans for assessment and collection of outcomes {18a}

At baseline, study staff will conduct interviews to capture baseline characteristics of the study sample, including demographics; exposure assessment (relationship with case patient; timing/intensity/frequency of contact; protective measures taken during contact); medical history (comorbidities, medications, smoking) and symptoms.

To obtain specimens for viral RNA testing (primary outcome), participants will be instructed on self-collection of oropharyngeal/nasal swabs as in prior work [64, 65], for RT-PCR testing [66–68]. Specifically, participants are asked to swab the back of their tongue, followed by the throat, before inserting the swab up to 4 cm into each nostril and rotating at least twice. These instructions are also provided in a short instructional video. Specimens will be collected at baseline, day 7, day 14 and within 24 h of new symptoms. In participants who are asymptomatic, a second self-collected swab will also be collected at day 14 and combined in the same specimen vial, or a saliva specimen will be collected if swabs are not available, since having two respiratory specimens tends to increase sensitivity for the detection of respiratory viruses [69, 70]. (If a participant has an earlier swab that is PCR positive then only one swab/saliva specimen needs to be collected at day 14.) Nasopharyngeal swabs and deep respiratory samples (e.g., bronchoalveolar lavage/endotracheal tube aspirates) already done for clinical care purposes will also be collected, with biobanking of specimens not tested in real-time for future analysis.

Follow-up interviews will be conducted at days 7, 14, 35, and 90 to assess for adverse events and clinical outcomes (hospitalizations, mechanical ventilation, mortality). Symptom data will be collected using daily electronic questionnaires that will be emailed daily for days 1–14. Participants will also be provided with a thermometer with which to record their temperature daily on days 1–14.

Methods for serologic testing of COVID-19 are still in development at the time of writing, but are rapidly emerging. At the baseline visit, participants in the active arm will self-collect dried blood spots for future testing for SARS-CoV-2 serology. These specimens will only be collected for the active arm since these participants will already be obtaining a finger-prick blood sample for HIV testing. All participants will undergo venipuncture at day 35 for batch testing of SARS-CoV-2 serology at the conclusion of the study. Self-collected saliva samples at days 1, 7, and 14 will also be used for antibody detection.

At day 14, participants will be asked to complete the K10, a short ten-item scale to measure non-specific psychological distress, as part of their electronic questionnaire. This rigorously developed instrument has differentiates between community cases and non-cases of mental health disorders according to the Structured Clinical Interview for DSM-IV [45, 46]. A standard cutoff score of ≥ 16 will be used to define short-term psychological distress.

The psychological impact of COVID-19 exposure will be assessed at day 90 using the Impact of Event Scale [47], a well-characterized 15-item scale has also been used in studies of health care workers exposed to pandemic H1N1 influenza [71, 72]. This instrument, which includes a seven-item intrusion subscale and eight-item avoidance subscale, was designed to measure the longer-term impacts of stressful life events and has good internal consistency, test-rest reliability, and sensitivity to change [47]. A standard cutoff score of ≥ 26 will be used to define a traumatic stress response [48]. Additional questions related to practical and functional impacts of COVID-19 exposure and used in previous studies on the impact of the SARS epidemic will be included at this time point also [48].

Multiple time points will incorporate the EQ-5D, a standardized instrument for assessing general health status and utility scores for incorporation into health economic analyses (https://euroqol.org/eq-5d-instruments/eq-5d-5l-about/). A utility score enables comparisons across different health interventions and diseases [73].

### Plans to promote participant retention and complete follow-up {18b}

At baseline, each participant will be asked to provide multiple modes of contact (e.g., telephone numbers, email addresses), as well as two additional persons that can be contacted in case the study team has difficulty reaching the participant for follow-up visits and clinical outcome ascertainment.

Access to serial SARS-CoV-2 testing from home will be an incentive to continued trial participation. Modest compensation will be provided in the form of $40 CAD at the day 35 in-person study visit, and another $10 CAD in the form of an electronic gift card after completion of the day 90 study visit in order to cover minor study-related costs (e.g., transportation/parking) and to encourage retention.

### Data management {19}

Study staff must maintain adequate and accurate source documents upon which case report forms for each participant are based. Data will be stored in an electronic study database (REDCap), with pre-programmed range checks to promote data quality. The trial data management center (Applied Health Research Centre of St. Michael’s Hospital) will conduct remote risk-based monitoring of this trial, including centralized review of essential study documents, as well as targeted source data verification of electronic data on 10% of participants, chosen at random.

### Confidentiality {27}

Participants will be identified using two unique study identification numbers only, and no identifying data will be stored in the central study database. No identifying information will be shared beyond the immediate members of the research team at the study site where the participant is enrolled. Participating sites are responsible for keeping any identifiable information in locked locations, if in hard copy, or in password-protected files on secure servers, if in soft copy.

### Plans for collection, laboratory evaluation, and storage of biological specimens for genetic or molecular analysis in this trial/future use {33}

Any respiratory, saliva, serum, and/or plasma specimens collected from participants but not analyzed in real-time will be stored in a biobank for potential future testing related to respiratory pathogen diagnostics and inflammatory biomarkers. No genetic testing of participants is planned.

## Statistical methods

### Statistical methods for primary and secondary outcomes {20a}

The primary outcome is microbiologically confirmed SARS-CoV-2 infection, i.e., detection of viral RNA in a respiratory specimen (oropharyngeal/nasal swab, nasopharyngeal swab, sputum specimen, saliva specimen, oral swab, endotracheal aspirate, bronchoalveolar lavage specimen) by day 14. Our primary analysis will be a generalized linear mixed model (GLMM) with logit link to estimate the effect of LPV/r on the probability of infection while accounting for clustering of participants in rings, with stratification by the randomizing site. The primary analysis will be unadjusted; in addition, multivariable models will adjust for key characteristics of both contacts (age, sex, co-morbidity, exposure characteristics such as type, duration, and timing) and index cases (illness severity, concomitant medications, etc.). In particular, we will include sex/gender-based analyses with variables specific to contacts and types of exposures. Participants with specimens positive for SARS-CoV-2 at baseline will be permitted to continue in the trial and follow all study procedures, including completion of the 14-day course of study drug for those in the active arm, as noted in section 12.2.2. These participants will be excluded from the primary and secondary analyses regarding LPV/r as PEP, but included in secondary analyses regarding early LPV/r treatment, as noted below.

To assess safety, adverse events will be tabulated according to grade and causality assessment using definitions from the DAIDS Table for Grading the Severity of Adverse Events [28], and compared between study arms.

As for the primary analysis outlined above, GLMM with logit link (for dichotomous outcomes) or identity link (for continuous outcomes) will also be used to compare the following secondary outcomes between study arms:

- Symptomatic COVID-19 disease by day 14
- Seropositivity by day 35
- Days of hospitalization attributable to COVID-19 disease by day 90
- Respiratory failure attributable to COVID-19 disease requiring (i) non-invasive and (ii) invasive ventilation by day 90
- Mortality attributable to COVID-19 disease
- Short-term psychological distress, defined as scoring ≥ 16 on the K10 [45, 46] at day 14
- Long-term traumatic stress response, defined as scoring ≥ 26 on the IES scale [47, 48] at day 90
- Health-related quality of life, measured by the EQ-5D-5 L on days 1, 14, 35, and 90

Additional secondary analyses will examine the role of LPV/r in early treatment of COVID-19, in the subset of participants whose baseline specimens test positive for SARS-CoV-2. GLMM models will be used to compare the same secondary outcome measures described above.

Key transmission-related epidemiologic parameters among exposed contacts including exposure histories, cluster size, secondary attack rate (p_0_, incidence proportion), time to first viral shedding will be analyzed using descriptive statistics. Risk factors for transmission and correlates of symptomatic disease will be analyzed using logistic regression models.

### Interim analyses {21b}

An interim analysis will be conducted when 122 clusters (50% of the planned number of clusters under the revised sample size calculation) have been followed-up for the primary outcome, adjusting the significance level according to Haybittle and Peto [74]. The Data Safety and Monitoring Committee will make recommendations at this point regarding whether to continue the trial as is or stop the trial for futility, with consideration of studying an alternative promising agent as COVID-19 PEP or otherwise altering the study intervention as applicable. The final decision regarding stopping or continuing the trial will rest with the Co-Principal Investigators, in consultation with the Steering Committee.

### Methods for additional analyses (e.g., subgroup analyses) {20b}

Subgroup analyses of the primary outcome will be performed according to the type of exposed contact (e.g., healthcare workers, household members) since prior data have suggested considerably different secondary attack rates for respiratory viruses in these settings; according to the number of days between exposure and enrollment, since PEP is most likely to be effective when started as soon as possible after exposure; and according to site/city, since small differences related to practice patterns and other unknown factors may exist.

### Methods in analysis to handle protocol non-adherence and any statistical methods to handle missing data {20c}

The primary analyses will be by intention-to-treat. Missing data will be imputed (e.g., regression, interpolation) under a variety of assumptions (e.g., missing at random, missing completely at random), and conduct sensitivity analyses to determine the robustness of our findings. Exploratory analyses will examine whether participants with greater adherence experience a lower risk of the primary outcome, using both self-report and pharmacokinetic methods (lopinavir levels in dried blood spots at day 14) to confirm adherence.

### Plans to give access to the full protocol, participant level-data and statistical code {31c}

The full study protocol is provided as an Appendix to this article. After the primary and secondary analyses have been completed, de-identified data will be uploaded to a public data repository (e.g., Dryad).

## Oversight and monitoring

### Composition of the coordinating center and trial steering committee {5d}

Day-to-day operations of the trial will be overseen by an experienced Project Manager at the lead site. The Steering Committee is composed of twelve individuals with expertise in infectious diseases, infection prevention and control, clinical trials, biostatistics, and medical microbiology, including the Co-Principal Investigators and Site Principal Investigators representing multiple cities.

Data management will be overseen by the Applied Health Research Centre at the Li Ka Shing Knowledge Institute of Unity Health Toronto, whose experienced project managers, research assistants, database developers, data managers, programmers, statistical team, and information technology staff currently support over 50 CIHR and NIH-supported clinical trials, registries, and observational studies.

### Composition of the data safety monitoring board, its role and reporting structure {21a}

The DSMC is an independent group of four individuals with expertise in clinical trials, infectious diseases, and biostatistics, appointed by and advisory to the Steering Committee. The DSMC will remain standing until the end of trial accrual. All DSMC members are expected to remain free from perceived or actual conflict of interest throughout their involvement in the trial and will be asked to complete a conflict of interest form at the start of their involvement in the DSMC. The DSMC Charter is posted at the study website (www.optionslab.ca/projects/coriprev) and is a living document that may be revised at regular intervals if needed.

Of note, the World Health Organization has established a process to promote data-sharing between individual clinical trials of COVID-19 pre-exposure prophylaxis, PEP, and treatment globally. After each review meeting, the CORIPREV-LR DSMB will confidentially share its closed report with DSMCs monitoring other, closely related clinical trials; the DSMCs for these other trials will do the same. This process will enhance the ability of each DSMC to provide optimal oversight to the individual trials regarding safety and efficacy without jeopardizing the integrity of each trial.

### Adverse event reporting and harms {22}

An adverse event (AE) is defined as any untoward medical occurrence in a patient or clinical investigation participant, administered a study medication/intervention, which does not necessarily have a causal relationship with this treatment. An AE can therefore be any unfavorable and unintended sign (including an abnormal laboratory finding), symptom, or disease temporally associated with the use of a medicinal (investigational) study medication/intervention, whether or not related to the medicinal (investigational) study medication/intervention.

During each follow-up visit with the participant, information on AEs will be gathered by study coordinators using open-ended questions about any untoward medical occurrences and documented accordingly. AEs will be graded as mild, moderate, severe, or life-threatening according to the DAIDS Table for Grading the Severity of Adverse Events [28] and assessed for causality as probably related, possibly related, unlikely to be related or not related to the study drug (investigational arm only). Data will be reported on AEs of any grade, whether expected or unexpected, regardless of causality assessment.

Serious adverse events (SAEs) are defined as AEs that are life-threatening; result in death, hospitalization, or prolongation of existing hospitalization; or result in significant disability or a congenital anomaly, with the exception of developing COVID-19, which will be captured as an outcome instead. SAEs must be reported to the Project Manager within 24 h of the site becoming aware of the event; the Project Manager will ensure prompt reporting to regulatory authorities.

### Frequency and plans for auditing trial conduct {23}

Trial monitoring will be conducted through a remote monitoring scheme including two components. First, an independent study monitor will conduct a centralized review of essential study documents at all participating sites related to participant protection, such as informed consent form signature pages, training records, delegation logs, and documentation of investigator qualifications. Second, targeted source data verification of critical data variables in the electronic case report forms will be performed on 10% of participants chosen at random.

### Plans for communicating important protocol amendments to relevant parties (e.g., trial participants, ethical committees) {25}

The Principal Investigators and Project Manager will be in continuous contact with investigators, site staff, ethics boards, and regulators about the trial and will notify all parties of relevant changes immediately, over the lifetime of the trial. Trial registries will be updated on a quarterly basis or more frequently, as relevant and appropriate. Actively enrolled participants will be notified of relevant changes to the protocol as soon as possible and no later than their next scheduled study visit, or immediately in the event of major safety or efficacy concerns.

## Dissemination plans {31a}

Given the urgency of disseminating findings in the context of the ongoing COVID-19 pandemic, results of the primary analysis will be shared as soon as possible with the broader scientific community through posting of our initial manuscript on a reputable pre-print server and rapid publication of results in a peer-reviewed journal. Participants will be encouraged to contact the study team directly or access the study website to learn about the results.

## Discussion

The COVID-19 pandemic has introduced an urgent need for rigorous clinical trials. There is a particularly pressing need for novel prevention tools. Worldwide, society remains in various states of strict isolation in large part because our primary prevention modalities include physical distancing and the use of barrier precautions such as face masks. Importantly, a variety of vaccines have now been demonstrated to have high efficacy in preventing COVID-19. However, vaccine coverage is likely to remain insufficient to control the pandemic in most global settings for many months or years. Key implementation challenges include manufacturing delays, distribution challenges, vaccine hesitancy, and the emergence of new SARS-CoV-2 variants [75–77]. Hence, ongoing research into alternative prevention modalities is still warranted, including the use of chemoprophylaxis. The CORIPREV-LR trial will generate high-quality evidence on whether a safe, existing drug prevents COVID-19 in close contacts of individuals with confirmed infection. If results are positive, LPV/r could immediately be deployed for this purpose, including in low-, middle- and high-income settings. Even if negative, the global availability of LPV/r means trial results will be impactful, by limiting off-label use.

An important consideration in the conduct of COVID-19 prevention trials is the need for vigilant infection control measures to limit the risk of onward transmission. To overcome this concern, this trial and others like it will conduct the majority of study interactions with participants remotely. While a minority of study visits may only be feasible through a telephone connection, the widespread availability of video-enabled smartphones and of secure platforms for online videoconferencing will allow most visits to be conducted through video-link. These measures will help to protect study personnel from potential exposure and facilitate participant observance of self-isolation directives, while still allowing study staff to visually ensure compliance with critical research procedures. In particular, the remote trial format also necessitates the use of specimen self-collection, including oropharyngeal/nasal swabs for SARS-CoV-2 testing and dried blood spots for serologic and pharmacokinetic assays. Our trial incorporates only a single in-person visit, at day 35, for the collection of venous blood samples for serologic testing.

To date, several clinical trials of COVID-19 PEP have already been completed and reported. An initial team of American and Canadian investigators randomized 821 asymptomatic adults who reported a household or occupational exposure to someone with COVID-19 within the preceding 4 days, to receive either hydroxychloroquine or placebo, and noted no statistically significant difference in the incidence or either laboratory-confirmed COVID-19 or a compatible illness over the subsequent 14 days [61]. A shortcoming of this trial was that the availability of microbiologic testing for outcome ascertainment was limited, such that infection was confirmed by polymerase chain reaction in only 14% of participants with symptomatic disease. Our trial will overcome this limitation by using specimen self-collection, and testing of these samples within 24 h for SARS-CoV-2. Two subsequent trials of hydroxychloroquine as COVID-19 PEP have similarly shown no impact of that drug on acquisition of SARS-CoV-2 infection, using microbiologically confirmed infection by PCR as the primary outcome [78, 79].

Another novel feature of our trial design is our inclusion of baseline testing for COVID-19 in all participants. This approach will further allow us to explore the impact of lopinavir/ritonavir in the early treatment of COVID-19, since those who test positive at baseline will continue all study activities. Despite high-quality trials demonstrating that lopinavir/ritonavir is not effective as treatment in hospitalized COVID-19 patients, questions about its efficacy in early-stage treatment and prophylaxis remain unanswered. Only one other clinical trial is examining the efficacy of lopinavir/ritonavir as COVID-19 PEP, using an individual-randomized design [80].

## Trial status

The CORIPREV-LR trial opened to recruitment on April 17, 2020, and anticipated to recruit for up to 18 months, depending on the evolution of the COVID-19 pandemic. At the time of writing, the trial is being conducted under protocol version 1.8, dated February 5, 2021.

## Supplementary Information

**Additional file 1: Appendix A.**

## Data Availability

The study team intends to make a de-identified dataset publicly available by submitting to a data repository after the main study analyses have been completed and published.

## Abbreviations

AE: Adverse event
CORIPREV: COVID-19 Ring-based Prevention trial
COVID-19: Coronavirus disease 2019
DAIDS: Division of AIDS
DSMC: Data Safety Monitoring Committee
GLMM: Generalized Linear Mixed Model
HIV: Human immunodeficiency virus
ICC: Intra-class correlation coefficient
LPV/r: Lopinavir/ritonavir
MERS-CoV: Middle East respiratory syndrome coronavirus
PCR: Polymerase chain reaction
PEP: Post-exposure prophylaxis
REDCap: Research Electronic Data Capture
RNA: Ribonucleic acid
RT-PCR: Reverse transcription polymerase chain reaction
SAE: Serious adverse event
SARS: Severe acute respiratory syndrome
SARS-CoV-2: Severe acute respiratory syndrome-related coronavirus 2
WHO: World Health Organization
