# Supplementary material for: Post-exposure prophylaxis against SARS-CoV-2 in close contacts of confirmed COVID-19 cases (CORIPREV): study protocol for a cluster-randomized trial
Source: Trials. 2021 Mar 22;22:224. doi: 10.1186/s13063-021-05134-7 (PMC7982877; doi:10.1186/s13063-021-05134-7)
Supplement: Supplementary file 1 — Additional file 1: Appendix A. [file 13063_2021_5134_MOESM1_ESM.pdf]

FULL TITLE:	COVID-19 Ring-based Prevention trial with Lopinavir/ritonavir<br>(CORIPREV LR)
Protocol No.	CORIPREV-1
Sponsor-Investigator-Investigator:	Dr. Darrell H. S. Tan
Co-Principal Investigators	Dr. Darrell H. S. Tan<br>Dr. Allison McGeer<br>Dr. Adrienne Chan
Study Support/Funding:	Canadian Institutes of Health Research<br>St. Michael`s Hospital Foundation<br>AbbVie
ClinicalTrials.gov Identifier:	NCT04321174
Version No.	1.8
Date:	5-February-2021

GCP Statement
This clinical study will be conducted in accordance with applicable Health Canada regulations, International Conference on Harmonisation of Technical Requirements for Registration of Pharmaceuticals for Human Use (ICH) guidelines on current Good Clinical Practice (GCP), and the Declaration of Helsinki.

Confidentiality Statement
This clinical study protocol contains information which is of a confidential, trade-secret or proprietary nature. The protocol is for the use of the Sponsor-Investigator and designated representatives participating in the investigational trial. It is not to be disclosed to any other person or party without the prior written approval of the Sponsor-Investigator.

TABLE OF CONTENTS

1. INVESTIGATOR AGREEMENT 5
2. STUDY CONTACT DETAILS 6
3. ABBREVIATIONS AND DEFINITIONS 7
4. PROTOCOL SYNOPSIS 9
5. INTRODUCTION, BACKGROUND, AND STUDY RATIONALE 12
   5.1 OVERVIEW 12
   5.2 COVID-19 BACKGROUND 13
   5.3 CURRENT TREATMENT OPTIONS AND RATIONALE FOR STUDYING LOPINAVIR/RITONAVIR 13
   5.4 POTENTIAL RISKS AND BENEFITS TO HUMAN PARTICIPANTS 15
   5.5 STUDY RATIONALE 15
6. STUDY OBJECTIVES AND DESIGN 16
   6.1 OVERALL STUDY DESIGN 16
   6.2 PRIMARY OBJECTIVE(S) 17
   6.3 SECONDARY OBJECTIVE(S) 17
   6.4 OUTCOME MEASURES 17
   6.5 SUB-STUDIES 18
7. SELECTION AND ENROLLMENT OF PARTICIPANTS 19
   7.1 NUMBER OF PARTICIPANTS 19
   7.2 INCLUSION CRITERIA 19
   7.3 EXCLUSION CRITERIA 20
   7.4 CONSIDERATIONS RELATED TO MEMBERSHIP IN MORE THAN ONE RING 21
   7.5 STRATEGIES FOR RECRUITMENT 23
   7.6 ENROLLMENT PROCEDURES 25
   7.7 CO-ENROLLMENT GUIDELINES 26
8. WITHDRAWAL OF PARTICIPANTS 266
   8.1 WITHDRAWAL CRITERIA 266
9. RANDOMIZATION AND BLINDING PROCEDURES 27
   9.1 RANDOMIZATION 27
   9.2 BLINDING 27
10. STUDY INTERVENTIONS 28
   10.1 LOPINAVIR/RITONAVIR 28
      10.1.1 Dosing and Administration 288
      10.1.2 Dosing in special populations 29
      10.1.3 Formulation and Packaging 299
      10.1.4 Storage and Stability 31
      10.1.5 Study Drug Dispensation 31
      10.1.6 Expected Side Effects 31
      10.1.7 Additional Safety Considerations 311
   10.2 CONTROL ARM 33
   10.3 CONCOMITANT AND PROHIBITED MEDICATIONS 33
   10.4 PARTICIPANT ACCESS TO STUDY MEDICATION AT STUDY CLOSURE 355
11. RISK MANAGEMENT 36

11.1 PREGNANCY 36
11.2 BREASTFEEDING 366
11.3 SUPPORTIVE MEDICATIONS 37
11.4 PROTECTION OF STUDY PERSONNEL FROM COVID-19 37

12. CLINICAL AND LABORATORY EVALUATIONS 38

12.1 CLINICAL EVALUATIONS 38
12.2 LABORATORY EVALUATIONS AND SPECIMEN COLLECTION 38
12.2.1 HIV Self-testing..38
12.2.2 Microbiologic testing for SARS-CoV-2 399
12.2.3 SARS-CoV-2 Serology..40
12.2.4 Oral microbiome analysis..40
12.2.5 Lopinavir levels..41
12.3 QUESTIONNAIRES 41
12.4 STORED RESEARCH SPECIMENS AND PLANS FOR POSSIBLE FUTURE TESTING 42

13. STUDY PROCEDURES 42

13.1 SCHEDULE OF EVENTS..42
13.2 SCREENING VISIT (DAY 0; CONDUCTED REMOTELY) 44
13.3 BASELINE VISIT (DAY 1; CONDUCTED REMOTELY UNLESS FEASIBLE IN-PERSON) 44
13.4 DAY 7 VISIT (CONDUCTED REMOTELY UNLESS FEASIBLE IN PERSON) 45
13.5 DAY 14 VISIT (CONDUCTED REMOTELY UNLESS FEASIBLE IN PERSON) 46
13.6 DAY 35 VISIT (CONDUCTED IN PERSON) 46
13.7 DAY 90 VISIT (CONDUCTED REMOTELY) 47
13.8 EARLY TERMINATION VISIT 48

14. EVALUATION, RECORDING, AND REPORTING OF ADVERSE EVENTS..48

14.1 DEFINITIONS..48
14.1.1 Adverse Event (AE) 48
14.1.2 Serious Adverse Events (SAEs)..48
14.2 AE DESCRIPTIONS AND RECORDING 49
14.3 FOLLOW-UP FOR ADVERSE EVENTS 50
14.4 PREGNANCY FOLLOW-UP 51

15. STATISTICAL CONSIDERATIONS..51

15.1 GENERAL STUDY DESIGN..51
15.2 SAMPLE SIZE CONSIDERATIONS/JUSTIFICATION 51
15.3 STATISTICAL ANALYSES 52
15.3.1 Analysis of Primary Outcome Measures 52
15.3.2 Analysis of Secondary Outcome Measures 52
15.3.3 Descriptive analyses..53
15.4 SUBGROUP ANALYSES..53
15.5 INTERIM ANALYSES 54

16. ETHICAL CONSIDERATIONS 54

16.1 ETHICAL CONDUCT OF THE STUDY..54
16.2 INFORMED CONSENT/ASSENT..54
16.3 CONFIDENTIALITY 55
16.4 INSTITUTIONAL REVIEW BOARD, ETHICS COMMITTEE, OR RESEARCH ETHICS BOARD..55

17. GENERAL TRIAL CONDUCT CONSIDERATIONS 56

17.1 ADHERENCE TO PROTOCOL..56
17.1.1 Protocol Amendments..56

17.1.2 Protocol Deviations ... *56*
17.2 MONITORING & AUDITING ... 56
17.2.1 Data Safety Monitoring Committee ... *56*
17.2.2 Study Monitoring ... *57*
17.3 RECORD KEEPING ... 58
17.3.1 Data Collection ... *58*
17.3.2 Source Documents ... *58*
17.3.3 Data Corrections ... *58*
17.3.4 Data Management ... *58*
17.3.5 Record Retention ... *59*
17.4 STEERING COMMITTEE ... 59
18. DISCLOSURE AND PUBLICATION POLICY ... 59
18.1 PUBLICATION OF STUDY RESULTS ... 59
18.2 DATA SHARING FOR SECONDARY RESEARCH ... 59
18.3 CONFLICT OF INTEREST ... 60
19. REFERENCES ... 61

1. INVESTIGATOR AGREEMENT

Protocol Title:	COVID-19 Ring-based Prevention trial with Lopinavir/ritonavir (CORIPREV LR)
Protocol No.:	CORIPREV-1
Version No.:	Version 1.8
Date:	5-February-2021

This clinical study will be conducted in accordance with applicable Health Canada regulations, ICH guidelines on current GCP, and the Declaration of Helsinki.

I confirm that I have read and understand this protocol and I agree to conduct this clinical study in accordance with the design and specific provisions of the protocol, with the exception of a change intended to eliminate an immediate hazard to participants. Any deviation from the study protocol will be documented in the case report form.

I agree to promptly report to the applicable ethics boards any changes in the research activity and all unanticipated problems involving risks to human participants or others. Additionally, I will not make any changes in the research without prior ethics and Sponsor-Investigator approval, except where necessary to ensure the safety of study participants.

Name	Signature	Date (dd-mmm-yyyy)

2. STUDY CONTACT DETAILS

Role	Contact Details
Principal Investigator,<br>Sponsor-Investigator	Darrell H. S. Tan, MD FRCPC PhD<br>Tel: 416-864-6060 x77505, Fax: 416-864-5310<br>darrell.tan@gmail.com
Co-Principal Investigators	Allison McGeer, MD FRCPC MSc<br>Tel: 416-586-2123, Fax: 416-586-3140<br>Allison.McGeer@sinaihealth.ca<br><br>Adrienne Chan, MD FRCPC MPH<br>Tel: 416-480-6100 x6044, Fax: 416-480-4268<br>adrienne.chan@sunnybrook.ca
Medical Monitor	Graham Smith, MD MSc CCFP<br>Tel: 416-465-0756, Fax: 416-465-8344<br>gsmith@mlmc.ca
Project Manager<br>SAE Reporting	Attia Qamar<br>Tel: 416-864-6060 x77325, Fax: 416-864-5302<br>Attia.Qamar@unityhealth.to
Study Pharmacists	Mark Naccarato, BScPharm<br>Tel: 416-864-6060 x 2697, Fax: 416-864-5310<br>Mark.Naccarato@unityhealth.to<br><br>Deborah Yoong, BScPhm, PharmD<br>Tel: 416-864-6060 x 6140, Fax: 416-864-5310<br>Deborah.Yoong@unityhealth.to
Data Coordination Centre	Applied Health Research Centre (AHRC)<br>Dominic Lee<br>Tel: 416-360-4000 x77058<br>Dominic.Lee@unityhealth.to
Clinical Laboratory Facility	Tony Mazzulli, MD FRCPC FACP<br>Tel: 416-586-4695, Fax: 416-586-8746<br>Tony.mazzulli@sinaihealthsystem.ca<br><br>Samira Mubareka, MD FRCPC<br>Tel: 416-480-4823, Fax: 416-480-6990<br>Samira.mubareka@sunnybrook.ca<br><br>Jennifer L. Gommerman, PhD<br>Tel: 416-978-6959 , Fax: 416-978-1938<br>Jen.gommerman@utoronto.ca

3. ABBREVIATIONS AND DEFINITIONS

Acronym / Abbreviation	Definition
ACTG	AIDS Clinical Trials Group
AE	Adverse Event
AHRC	Appied Health Research Centre
ALT	Alanine aminotransferase
CADTH	Canadian Agency for Drugs and Technologies in Health
CATCO	CAnadian Treatments for COvid19
COVID-19	Disease caused by the 2019 Novel Coronavirus, also known as 2019-nCoV or SARS-CoV-2
CRF	Case Report Form
CYP	Cytochrome P450
DAIDS	Division of AIDS, National Institutes of Health
DBS	Dried Blood Spot
DIN	Drug Identification Number
DSMC	Data Safety Monitoring Committee
eCRF	Electronic Case Report Form
EDC	Electronic Data Capture
EQ-5D-5L	EuroQol 5 Dimension 5 Level scale
FFX	First Few X
GCP	Good Clinical Practice
GLMM	Generalized Linear Mixed Model
HCW	Health Care Worker
HIV	Human Immunodeficiency Virus
ICC	Intraclass Correlation Coefficient
ICER	Incremental Cost-Effectiveness Ratio
ICF	Informed Consent Form
ICH	International Conference on Harmonization
IES	Impact of Event Scale
IPAC	Infection Prevention and Control
GLMM	Generalized Linear Mixed Models

HCV	Hepatitis C Virus
LPV/r	Lopinavir/ritonavir
MERS	Middle East Respiratory Syndrome
NP	Nasopharyngeal
p_0	Secondary attack rate
PAH	Pulmonary arterial hypertension
PCR	Polymerase Chain Reaction
PDE5	Phosphodiesterase 5
PEP	Post-exposure prophylaxis
PHAC	Public Health Agency of Canada
PI	Protease Inhibitor
PPE	Personal Protective Equipment
PUI	Person Under Investigation
QALY	Quality Adjusted Life Year
RCT	Randomized controlled trial
REB	Research Ethics Board
REDCap	Research Electronic Data Capture
RNA	Ribonucleic acid
RR	Relative Risk
RT-PCR	Reverse Transcription Polymerase Chain Reaction
SAE	Serious Adverse Event
SARS	Severe Acute Respiratory Syndrome
SCID	Structured Clinical Interview for DSM-IV
TCPS2	2nd edition of the Tri-Council Policy Statement: Ethical Conduct of Research in Humans
WHO	World Health Organization

4. PROTOCOL SYNOPSIS

Full Title	COVID-19 Ring-based Prevention trial with Lopinavir/ritonavir
Short Title	CORIPREV LR
Protocol Number	CORIPREV-1
Version Number and Date	Version 1.8, February 5, 2021
Clinical Phase	III
Study Duration	Enrollment period: April 6, 2020 to September 30, 2021
	Study period: April 6, 2020 to September 30, 2022
Sponsor-Investigator	Darrell H. S. Tan
Number of Centres	6 sites in Canada, with potential to expand further
Study Design	Open-label cluster-randomized trial
Primary Objective	To evaluate the efficacy of a 14-day course of oral lopinavir/ritonavir (LPV/r) as PEP against microbiologically confirmed SARS-CoV-2 infection among individuals with a significant unprotected exposure to a confirmed case.
Secondary Objectives	1. To compare the following secondary outcomes between study arm:<br>a) safety;<br>b) symptomatic COVID-19 disease by day 14;<br>c) seropositivity to SARS-CoV-2 by day 35;<br>d) hospitalization attributable to COVID-19 disease by day 90<br>e) respiratory failure attributable to COVID-19 disease requiring i) non-invasive ventilation or ii) intubation/mechanical ventilation by day 90;<br>f) mortality attributable to COVID-19 disease by day 90;

	g) short-term psychological distress associated with COVID-19 exposure at day 14<br>h) long-term psychological distress associated with COVID-19 exposure at day 90;<br>i) Health-related quality of life.<br><br>2. To characterize key transmission-related epidemiologic parameters among exposed contacts in a Canadian context, including exposure histories, cluster size, secondary attack rate (p_0, incidence proportion), time to first viral shedding and burden, risk factors for transmission, and correlates of symptomatic disease.
Sample Size	Preliminary target: 244 clusters of approx. 5 individuals per cluster (total N = 1220)
Randomization	1:1 cluster randomization to LPV/r vs no intervention, stratified by study site
Study Population	Non-vaccinated individuals 6 months and older who, within the past 1-7 days, have had high-risk close contact with a confirmed COVID-19 case during their symptomatic period, including one day before symptom onset. High-risk close contact is defined in accordance with guidance from the Public Health Agency of Canada, as any of the following exposures without the consistent appropriate use of recommended personal protective equipment:<br>a) Provided direct care for the index case<br>b) Had close physical contact with the index case<br>c) Lived with the index case<br>d) Had indoor close contact (within 2 metres), with or without direct physical contact, for at least one hour<br>e) Had direct contact with infectious body fluids, including oral secretions, respiratory secretions, or stool.
Investigational Product Description	Lopinavir/ritonavir (Kaletra® or Aluvia®)<br>200/50 mg tablets (Kaletra® DIN:02285533)

	80/20 mg/mL oral solution (Kaletra® DIN:02245644)
Control	No intervention
Administration and Dosing	400/100 mg (two 200/50 mg tablets) orally twice daily or equivalent weight-based dosing
Duration of Treatment	14 days
Outcome Measures	Primary: Microbiologically confirmed SARS-CoV-2 infection, ie. detection of viral RNA in a respiratory specimen (oropharyngeal/nasal swab, nasopharyngeal swab, sputum specimen, saliva specimen, oral swab, endotracheal aspirate, bronchoalveolar lavage specimen) by day 14.
	Secondary:<br>a) Adverse events<br>b) symptomatic COVID-19 disease<br>c) Seropositivity to SARS-CoV-2 at day 35<br>d) Hospitalization attributable to COVID-19<br>e) Respiratory failure attributable to COVID-19 requiring i) non-invasive ventilation or ii) endotracheal intubation with ventilation at day 90<br>f) Mortality attributable to COVID-19<br>g) Short-term psychological impact at day 14<br>h) Long-term psychological impact at day 90<br>i) Health-related quality of life
Statistical Analysis	The primary analysis will be a generalized linear mixed model (GLMM) with logit link to estimate the effect of LPV/r on the probability of infection while accounting for clustering of participants in rings. Multivariable models will adjust for key characteristics of both contacts (age, sex, co-morbidity, exposure characteristics) and index cases (illness severity, concomitant medications etc.).

5. INTRODUCTION, BACKGROUND, AND STUDY RATIONALE

5.1 Overview

Despite ongoing global efforts to contain COVID-19,[1-4] it has rapidly evolved into a global pandemic due to the lack of population immunity, growing evidence of undiagnosed community spread, and basic reproductive number of ~ 2.5 in China.[5] Canada is likely to see a considerable number of cases arising outside of China, Iran and Italy, due to strong historical, demographic, economic and travel ties, and was among the first countries to report a COVID-19 case.[6,7] As of March 17, 2020, there are just over 400 confirmed cases in Canada, and definitive evidence of local transmission.

Given the relative absence of evidence-based therapies for those with established disease, large numbers of hospitalizations and ICU admissions, and case fatality of up to 2.1% (95% CI 0.5-5.4%),[8] the prevention of new cases is critical. Importantly, a variety of vaccines have now been demonstrated to have high efficacy in preventing COVID-19.[9,10] However, vaccine coverage is likely to remain insufficient to control the pandemic in most global settings for many months or years. Key implementation challenges include manufacturing delays, distribution challenges, vaccine hesitancy, and the emergence of new SARS-CoV-2 variants.[11-13] Prevention measures based purely on isolating patients with suggestive symptoms and/or a relevant exposure history will be inadequate to control the spread of disease given that transmission can occur from mild and subclinical cases,[14,15] and given that even asymptomatic transmission may occur.[16,17] Hence, ongoing research into alternative prevention modalities is still warranted, including the use of chemoprophylaxis. Developing strategies for post-exposure prophylaxis (PEP) was thus identified as an urgent research priority at a recent Blueprint R&D forum convened by the WHO regarding the epidemic. This approach has already been attempted for the closely related Middle East respiratory syndrome coronavirus (MERS-CoV), with observational data suggesting a potential decrease in infection.[18] This protocol outlines a randomized, open-label cluster trial (RCT) of PEP against COVID-19 among exposed individuals, using a novel ring prophylaxis design. The trial includes an adaptive design to modify the target sample size according to emerging data.

Lopinavir/ritonavir (LPV/r, marketed in Canada as Kaletra®, and in some other markets as Aluvia®) is a promising antiviral agent that is ideally suited for study as PEP against COVID-19, because molecular, animal model and early clinical data suggest it to have activity against closely related coronaviruses (section 5.3). There is extensive experience using this agent in both the treatment and prevention of HIV,[19-21] and it has a well-established safety profile such that treatment-limiting adverse events and organ damage are rare. This trial will address the immediate need for preventive interventions, and further serve as a research platform anticipating candidate vaccines and other prevention modalities.

In addition, there are important unknowns about how transmission occurs that require urgent answers to inform ongoing prevention and mitigation strategies. For instance, we need data on route of transmission (role of respiratory droplet, airborne, formites, fecal-oral routes), estimates for the risk to individuals exposed to an index case (attack rate) and sources of heterogeneity in attack rates, and understanding on the correlation between viral shedding (timing, duration, intensity) and development of disease among both contacts and in those with early subclinical infection.[22] By collecting detailed epidemiologic, clinical and microbiologic data on exposed contacts, this trial will allow us to evaluate the transmissibility and natural history of this novel disease amongst exposed contacts in the Canadian setting. Data collection processes will be harmonized with protocols established by the World Health Organization,[23-25] and findings pooled with international consortiums to maximize comparability and impact.

5.2 COVID-19 Background

The history of COVID-19 shows how quickly a new zoonotic disease can develop into a global pandemic in the absence of human immunity, and underscores the need for a rapid research response focused on prevention. In December 2019, sporadic cases of fatal pneumonia were diagnosed in individuals with links to the Huanan seafood market in Wuhan, a city of 11 million in Hubei province, China. Local scientists rapidly isolated the responsible pathogen, a novel coronavirus with homology to bat and pangolin isolates,[26-28] and case series described its clinical features: rapid progression to respiratory failure, bilateral interstitial infiltrates on computed tomography of the chest, and a case fatality rate of 15% among the first 41 patients hospitalized with the disease.[3]

Spread of the epidemic progressed rapidly. By Feb 25, 2020, the number of new cases diagnosed outside China exceeded those within it.[29] In the absence of targeted control measures, explosive outbreaks rapidly emerged in other settings: Iran reported a total of 978 cases just 11 days after its first diagnosis and Italy reported 1689, with a rapid rise just 10 days after indications of community spread in Lombardy.[30] Canada was among the first countries to report a case outside China,[7] and community transmission of the virus was detected in Canada by early March 2020.

5.3 Current Treatment Options and rationale for studying lopinavir/ritonavir

There are few agents with proven, clinically relevant activity against COVID-19 in humans. Initially, there were several agents with biological and clinical plausibility that warranted further study.[31] Among these were remdesivir, a novel, broad-spectrum nucleotide analog with activity against multiple RNA viruses.[32] In a rhesus macaque model of MERS-CoV, remdesivir pre-exposure prophylaxis afforded protection against clinical infection, and decreased viral loads compared to control; PEP within 12 hours after inoculation also reduced viral loads and attenuated clinical signs of infection.[33] The anti-malarial chloroquine had also been shown to have antiviral activity against SARS-

CoV-2 in vitro, as had remdesivir.[34] Quercetin is a plant-derived flavonoid with in vitro antiviral effects.[35] Angiotensin converting enzyme inhibitors (ACE-i), an existing class of cardiovascular drug, have been considered because they target the receptor used by COVID-19 for cell entry.[36,37] However, subsequent clinical trials have failed to demonstrate clinically meaningful antiviral activity of these agents in vivo.[38]

Lopinavir/ritonavir was selected for this trial based on preclinical data suggesting it to be particularly promising anti-COVID-19 agent, its well-established safety profile, its feasibility for use in this trial, and its widespread availability. Prior molecular data show it to have activity against the SARS-CoV protease M^{pro}.[39] *In vitro* data show that it inhibited MERS-CoV viral activity in a low micromolar range in cell culture.[40] In an animal model, LPV/r led to better clinical, radiological and pathological outcomes as well as reduced mortality in marmosets infected with MERS-CoV.[41]

Encouragingly, a small observational study conducted among healthcare workers (HCW) in the context of a hospital-based outbreak of MERS-CoV in South Korea suggested protective efficacy of an 11 to 13-day course of LPV/r at a dose of 400/100 mg every 12 hours in combination with 6-8 days of ribavirin.[18] In that report, 0/22 (0%) of HCW receiving PEP acquired infection at 11 days after treatment initiation, whereas a retrospective review of records from four other hospitals with super-spread events showed that 6/21 (28.6%) of HCW who did not receive PEP acquired infection, giving an odds ratio of 0.405 (95%CI=0.274,0.599). In adjusted analyses, PEP was the only factor associated with reduced risk of MERS-CoV infection (OR=0.714, 95%CI=0.545, 0.936); the report does not specify what other factors were controlled for. However there are several limitations of this study that preclude a definitive conclusion about the potential efficacy of LPV/r PEP against COVID-19 disease, including the non-randomized nature of the study, the lack of standardization in PEP regimen, the small sample size, the relatively short follow-up time, and the lack of clarity regarding some of the statistical analyses.

In addition, for many years, LPV/r has been a key component of HIV treatment[20] and HIV PEP regimens.[21] The drug is widely available worldwide, including in resource-limited settings, with high quality manufacturing and supply chains. As a result, interest in and feasibility of LPV/r as COVID-19 PEP will be high as the epidemic expands. For these reasons, it is critical to formally evaluate the efficacy of LPV/r as PEP against COVID-19 using a rigorous randomized trial.

Importantly, the study team will also closely monitor emerging findings from ongoing treatment trials, including 319 trials already registered in China,[42] and consider adapting the choice of study drug if an alternative agent appears more promising at study initiation. Any such modification would require a formal amendment to the study protocol, regulatory approvals, and ethical approvals.

An initial clinical trial in China found no definite treatment benefit of LPV/r in hospitalized COVID-19 patients,[43] but was underpowered for the primary endpoint of mortality and initiation of LPV/r was late in the course of COVID-19 disease. There was also a

potential for imbalance in baseline characteristics (higher viral load, later presentation, more severe illness in the LPV/r arm) to influence the outcomes. Secondary endpoints hinted at potential benefit (numerically lower mortality, shorter ICU/hospital stay).[44] As noted by many observers,[45-49] the results underscore the need for more LPV/r trials. While subsequent results from the SOLIDARITY and RECOVERY trials showed no impact of treatment with LPV/r on mortality in hospitalized COVID-19 patients,[38,50] data are lacking on the impact of this drug when used for prophylaxis or pre-emptive therapy.

5.4 Potential Risks and Benefits to Human Participants

LPV/r is a safe, well-characterized antiviral. It is safe in pregnancy, liver disease and all levels of renal function including dialysis, and is available in liquid forms, facilitating use in children and intubated patients. Though a potent CYP3A4 inhibitor, drug interactions are well characterized; few medications are absolutely contraindicated, and dose adjustments are established for a wide range of other drugs based on pharmacokinetic studies.[51] Its main tolerability-related limitation is its potential for gastrointestinal (GI) upset; moderate/severe diarrhea, nausea and abdominal pain occurred in up to 28%, 16% and 11% respectively of adults taking LPV/r together with two other anti-HIV drugs in clinical trials.[52-55] Hyperlipidemia also occurs, but only after months of use rather than the 14 days to be used in this trial. Clinical trials using 28 days of LPV/r-based HIV PEP have shown GI side effects in ~half of participants, although discontinuations for adverse events were uncommon.[56-58] Overall, the safety of LPV/r suggests that the potential benefit to human participants exposed to a confirmed case of COVID-19 generally outweighs potential risks.

5.5 Study Rationale

The research response to COVID-19 has disproportionately ignored prevention interventions. While the global research community mobilized rapidly in response, and although the WHO has declared prophylaxis studies a priority, the overwhelming majority of interventional studies are focused on evaluating treatment interventions for those with confirmed disease. As of Mar 2, 2020, the Chinese Clinical Trials Registry lists 319 studies of which there are only 5 prevention trials, and of those only one tests a drug (umifenovir); clinicaltrials.gov has 54 coronavirus related intervention trials, and only one prevention study looking at traditional Chinese medicine. We are aware of only one other COVID-19 PEP trial, using a different drug (hydroxychloroquine).

Prevention interventions are critical for the trial's key target populations. We expect three main groups of exposed contacts, each with high anticipated acceptability for this trial. The most vulnerable patients in respiratory viral outbreaks are typically the frail elderly in congregate living or healthcare facilities due to difficulty controlling transmission in this setting and more severe consequences of infection. If COVID-19 becomes endemic, current estimates of the reproductive ratio in closed settings suggest

that impact on nursing/retirement homes will mimic influenza in the pre-vaccine/prophylaxis era, when mortality was ~20% or more.[59]

Healthcare workers (HCW) are also key, comprising 2% of COVID-19 cases in China and up to 29% of hospitalized patients.[1,60] The 2003 Canadian experience with SARS, in which 35% of cases and 7% (3/44) of deaths were in HCW[61,62] may drive higher anxiety regarding COVID-19 among HCW. Nosocomial amplification of illness has also been a prominent element of MERS-CoV outbreaks.[63] In addition, prior data show that most HCW (94.6%) with acute respiratory illness continue to work while symptomatic for a variety of reasons, and thus may inadvertently expose their patients to infection.[64] In the face of an epidemic, it is vital that HCW needs be met, to maintain morale among staff,[65] ensure a functional workforce, and protect patients.

The final group is household/community contacts. Breaking chains of community spread will be vital to limiting the impact of this disease. Given the media attention regarding COVID-19, this group will also likely have high interest in a prevention trial.

6. STUDY OBJECTIVES AND DESIGN

6.1 Overall Study Design

This study is a randomized, open-label cluster trial of oral LPV/r 400/100 mg twice daily as PEP to prevent SARS-CoV-2 infection among individuals with unprotected exposures (high-risk close contacts) to a confirmed COVID-19 case. High-risk close contact is as defined in section 7.2 and in accordance with guidance from the Public Health Agency of Canada.[66] The trial will use a ring-based approach to delivering these preventive strategies, adapting a novel cluster RCT design recently used in the 2013-16 West African Ebola epidemic in the *Ebola ça suffit* study.[67,68] The approach is to define a ring of exposed contacts around any newly confirmed COVID-19 case (herein referred to as the 'index COVID-19 case' for that ring), and to randomize rings (ie. clusters) to the study drug or no intervention.[69] Ring vaccination was key to the successful eradication of smallpox.[70] The rationale for a ring design in the Ebola trial was twofold. First, it maximizes statistical power by recruiting those at highest risk of infection; second, it seeks to limit transmission by creating a buffer of immune persons around cases. Both considerations apply to COVID-19, for which the urgent need for a response and significant consequences of infection demand an efficient, rigorous trial design with maximum potential to limit transmission. Another advantage is that the SARS experience predicts that some COVID-19 cases may lead to "super-spreading" events, with unusually high numbers of secondary cases.[71] This variability is taken into consideration by treating index cases, rather than exposed contacts, as the unit of randomization.

Participants will be seen at baseline, and then undergo follow-up at day 7, 14, 35 and 90. The primary outcome variable is microbiologically confirmed SARS-CoV-2 in a

respiratory specimen by day 14. In accordance with adaptive design principles,[72,73] a preliminary analysis will be conducted at the interim analysis to estimate the secondary attack rate (p_0) and intra-cluster correlation coefficient (ICC) for the primary outcome measure; these values will be used to re-assess the sample size calculations. An interim analysis will be conducted when 122 clusters have been followed-up for the primary outcome, adjusting the significance level according to Haybittle and Peto.[74]

6.2 Primary Objective(s)

The primary objective of this trial is to evaluate the efficacy of a 14-day course of oral lopinavir/ritonavir (LPV/r) as PEP against microbiologically confirmed SARS-CoV-2 among individuals with a significant unprotected exposure to a confirmed case.

6.3 Secondary Objective(s)

Secondary objectives are

1. To compare the following secondary outcomes between study arm:
   a) safety;
   b) symptomatic COVID-19 disease;
   c) seroconversion;
   d) hospitalization
   e) respiratory failure requiring i) non-invasive ventilation or ii) intubation/mechanical ventilation;
   f) mortality;
   g) short-term psychological distress associated with COVID-19 exposure;
   h) long-term psychological distress associated with COVID-19 exposure;
   i) health-related quality of life.

2. To characterize key transmission-related epidemiologic parameters among exposed contacts in a Canadian context, including exposure histories, cluster size, secondary attack rate (p_0, incidence proportion), time to first viral shedding and burden, risk factors for transmission, and correlates of symptomatic disease.

6.4 Outcome measures

The **primary outcome is microbiologically confirmed SARS-CoV-2 infection**, ie. detection of viral RNA in a respiratory specimen (oropharyngeal/nasal swab, nasopharyngeal swab, sputum specimen, saliva specimen, oral swab, endotracheal aspirate, bronchoalveolar lavage specimen) by day 14 of the study.

Secondary outcomes are:

a) **Adverse events:** as defined using the DAIDS Table for Grading the Severity of Adverse Events, at 7, 14, 35 & 90 days.[75]

b) **Symptomatic COVID-19 disease**: fever, cough or other respiratory/ systemic symptoms (including but not limited to fatigue, myalgias, arthralgias, shortness of breath, sore throat, headache, chills, coryza, nausea, vomiting, diarrhea) by day 14 in a patient with laboratory confirmed infection, combined with microbiologic confirmation of SARS-CoV-2 infection in the participant.

c) **Seropositivity:** reactive serology at day 35

d) **Hospitalization attributable to COVID-19 disease:** This will be considered both as a dichotomous outcome and as the number of days (or partial days) spent admitted (or not admitted) to an acute care hospital will be tabulated both at day 35 and day 90.

e) **Respiratory failure requiring ventilatory support attributable to COVID-19 disease:** The number of days (or partial days) requiring i) non-invasive and ii) endotracheal intubation with ventilation will be tabulated both at day 35 and day 90.

f) **Mortality** attributable to COVID-19 disease and all-cause mortality will be tabulated at 35 & 90 days.

g) **Short-term psychological impact of exposure to COVID-19** will be measured at day 14 using the K10, a validated measure of non-specific psychological distress, with a standard cutoff score of ≥16.[76,77]

h) **Long-term psychological impact of exposure to COVID-19** will be measured at day 90 using the Impact of Event Scale, a validated measure of traumatic stress response, using a standard cutoff score of ≥26.[78,79]

i) **Health-related quality of life** will be measured using the EQ-5D-5L (EuroQol-5D).[80] The EQ-5D consists of two pages: the EQ-5D descriptive system and the EQ visual analogue scale (EQ VAS). The descriptive system comprises five dimensions: mobility, self-care, usual activities, pain/discomfort and anxiety/depression. The tool will be administered to participants at 1, 14, 35 and 90 days.

6.5 Sub-studies

We will conduct two types of health economic evaluations: 1) cost-effectiveness analysis and 2) budget impact analysis. First, from the perspective of the healthcare

system, we will estimate the incremental cost-effectiveness ratio (ICER) per COVID-19 case prevented at 14 days attributable to LPV/r-based PEP in parallel with the intention-to-treat analyses for the primary outcome. We will also estimate the ICER for key secondary outcomes, including quality-adjusted life years (QALYs) in a cost-utility analysis using the EQ-5D data, attributable hospitalization and attributable mortality at 90 days. The cost-effectiveness analyses will follow Canadian and international modeling guidelines and best practices.[81,82] Parameter uncertainty will be examined through probabilistic analysis. Methodological uncertainty will be examined through scenario analyses.

Second, we will conduct a budget impact analysis from the public healthcare payer perspective, using trial results. Costs will include per-visit costs of clinical visits, lab tests and study drug. We will prospectively collect cost data on study drugs, health services (eg. laboratory tests, clinic visits) and assessment time (including telephone-based assessments) associated with the intervention and control strategies. Cost data will come from applicable provincial fee schedules for outpatient physician and laboratory testing services, and the provincial wholesale purchase costs for study drug. Additional costs will be drawn from the published literature using Canadian data. We will use these data to estimate the cost per patient of PEP compared to no intervention, over a 5-year time horizon, using guidelines by the International Society for Pharmacoeconomics and Outcomes Research Task Force.[83] A simple budget impact analysis focused solely on drug costs will also be conducted.

All participants in the primary study will be included in the economic sub-studies, and no additional participant enrollment procedures are needed.

7. SELECTION AND ENROLLMENT OF PARTICIPANTS

7.1 Number of Participants

The trial will enroll roughly 244 clusters of exposed high-risk close contacts at sites in Canada. Initially, the trial will include a limited number of sites in Toronto and then Vancouver only. The number of included sites will be re-evaluated as the epidemic evolves, with additional sites considered for initiation according to study progress and the availability of cases.

7.2 Inclusion Criteria

1. Within the past 1-7 days, high risk close contact with a confirmed COVID-19 case. If the index case was symptomatic, this contact must have occurred during their symptomatic period, including one day before symptom onset. If the index

case was asymptomatic, this contact must have occurred within 14 days of the index case's first positive SARS-CoV-2 test. High risk close contact is defined as any of the following exposures without the consistent appropriate use of recommended personal protective equipment:

a. Provided direct care for the index case
b. Had close physical contact with the index case
c. Lived with the index case
d. Had close indoor contact (within 2 metres), with or without direct physical contact, for at least one hour
e. Had direct contact with infectious body fluids, including oral secretions, respiratory secretions, or stool.

This definition is in accordance with the definition of a high-risk close contact for COVID-19 set out by the Public Health Agency of Canada.[66]

2. Successfully contacted by the study team within 48 hours of study team notification of the relevant index COVID-19 case. This time window is necessary because the efficacy of PEP may be dependent on the timing of its initiation, and because randomization of a ring cannot be delayed while awaiting response from contacts that cannot be rapidly reached.

3. Age ≥6 months, since the safety and pharmacokinetic profiles of LPV/r in pediatric patients below the age of 6 months have not been established.

4. Ability to communicate with study staff in the language(s) of the study site

It is expected that contacts will be classified into three main groups: those exposed in congregate living or healthcare facilities, healthcare workers (HCW), and community contacts.

7.3 Exclusion Criteria

1. Known hypersensitivity/allergy to lopinavir or ritonavir.

2. Current use of LPV/r for the treatment or prevention of HIV infection.

3. Receipt of LPV/r in the context of this trial or any other trial of COVID-19 PEP within 2 days or less prior to the last known contact with the index COVID-19 case. The two day time window is intended to ensure that exposure would not have occurred in the presence of clinically relevant drug levels (five times the elimination half-life of LPV/r, which is estimated at 4-6 hours with prolonged use).

4. Already known to be positive for COVID-19.

5. Currently breastfeeding an infant, due to potential for serious adverse reactions in nursing infants exposed to variable levels of LPV/r in breastmilk.

6. Concomitant medications with prohibited drug interactions with LPV/r that cannot be temporarily suspended/replaced, including but not restricted to: [51]

   - alfuzosin (e.g. Xatral®)
   - amiodarone (e.g. Cordarone™)
   - apalutamide (e.g. Erleada™)
   - astemizole*, terfenadine*
   - cisapride*
   - colchicine, when used in patients with renal and/or hepatic impairment
   - dronedarone (e.g., Multaq®)
   - elbasvir/grazoprevir (e.g., Zepatier™)
   - ergotamine* (e.g. Cafergot®*), dihydroergotamine (e.g. Migranal®), ergonovine, methylergonovine*
   - fusidic acid (e.g., Fucidin®), systemic*
   - lurasidone (e.g., Latuda®), pimozide (e.g., Orap®*)
   - neratinib (e.g., Nerlynx®)
   - sildenafil (e.g., Revatio®)
   - triazolam (e.g. Halcion®), midazolam oral*
   - rifampin (e.g. Rimactane®*, Rifadin®, Rifater®*, Rifamate®*)
   - St. John's Wort
   - Tadalafil (e.g. Cialis®, Adcirca®)
   - venetoclax (e.g. Venclexta®)
   - lovastatin (e.g., Mevacor®*), lomitapide (e.g., Juxtapid™) or simvastatin (e.g., Zocor®)
   - vardenafil (e.g., Levitra® or Staxyn®)
   - salmeterol (e.g., Advair® or Serevent®)

   **denotes products not marketed in Canada*

7. Receipt of any doses of any locally licensed COVID-19 vaccine.

Individuals who have been previously enrolled in this trial are permitted to re-enroll as long as they do not meet exclusion criterion #3.

7.4 <u>Considerations related to membership in more than one ring</u>

It is possible that one individual may be identified as part of more than one ring either sequentially or even simultaneously. The approach taken in these situations should be based on the temporal sequence in which study staff first make contact with the individual, and not the timing of the person’s exposure to the relevant index cases.

Sequential notification: If notification of this situation to the participant occurs sequentially, and the participant's initial ring had already been randomized to the control situation (no intervention), then for ethical reasons, the participant should be given the choice of whether to re-enroll in the second ring, since this re-enrollment may provide the opportunity to be randomized to the study drug. The decision regarding re-enrollment in the second ring must be made prior to randomization of that second ring; once randomization has occurred the participant cannot switch their ring affiliation. If the participant is already taking study drug at the time they are notified about membership in a second ring, then they are not eligible to re-enroll in the second ring. If the participant is not currently taking study drug at the time of notification, they may be eligible to re-enroll, but only if they were not taking study drug within two days of their exposure to the second index case. A flowchart is provided in Figure 1.

Simultaneous notification: If a participant is simultaneously notified about membership in two or more rings, the participant should be offered the choice of which ring they wish to be affiliated with. This decision must be made prior to randomization of either ring. It is anticipated that such decisions may be based purely on convenience considerations (eg. a participant may wish to be affiliated with the same ring as other family members).

Figure 1. Flowchart for handling membership in multiple rings

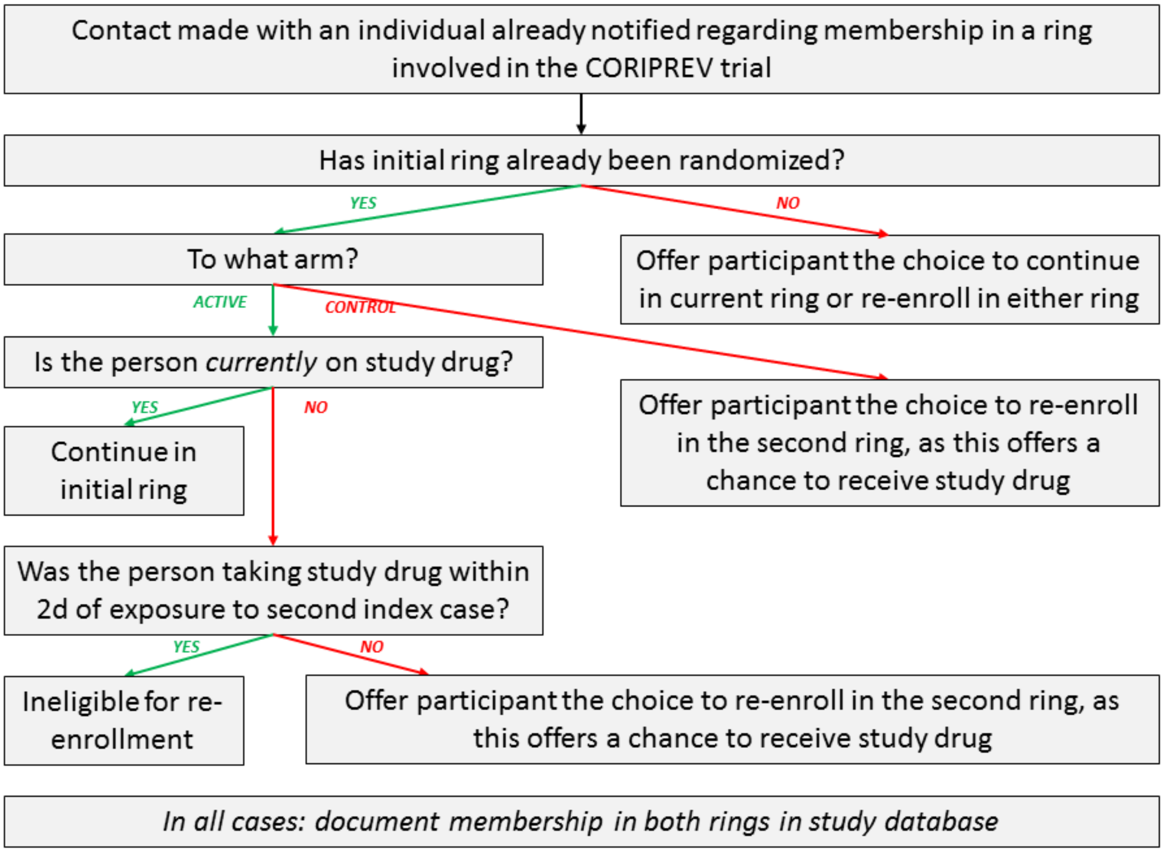

In all cases, information about membership in multiple rings must be captured in the study database.

7.5 Strategies for Recruitment

Enrollment of exposed persons requires initial identification of new lab-confirmed COVID-19 cases. We will **define a ring of exposed contacts around index cases**, using the definition of close contacts recommended by the World Health Organization (WHO; section 7.2). The study team will actively seek out such new cases through several mechanisms (Figure 2). Importantly, since many individuals who eventually go on to have a confirmed diagnosis will initially be deemed a "Person Under Investigation" (PUI) when they first undergo testing, then in order to provide extra time for contacting potential participants and allowing them to review the informed consent forms, we will apply the strategies below both for newly confirmed cases, as well as PUIs. It will be emphasized to the contacts of PUIs that they will only be eligible for enrollment if the index case's diagnosis is microbiologically confirmed. If these contacts agree to potential participation, then in the event that the PUI's diagnosis is later microbiologically confirmed, it is possible that the study team may subsequently confirm the index case's diagnosis with them prior to the index case directly communicating this information to the contact personally. For this reason, PUIs must provide consent to this possibility before the study team speaks with the close contacts regarding enrolment.

First, the study team is in regular contact with leaders of hospital infection prevention and control (IPAC) units from across the Toronto and Vancouver areas. In the event of a PUI/new case being identified, the IPAC teams may alert the study team without disclosing any personal information about the case in order to help the study team prepare for rapid enrollment activities, and simultaneously conduct contact tracing as per routine procedures. In the course of conducting contact tracing, the IPAC team will ask exposed contacts for permission to provide their name and contact information to study staff; if provided, the study team will contact the individual to initiate recruitment activities.

Figure 2. Recruitment flowchart

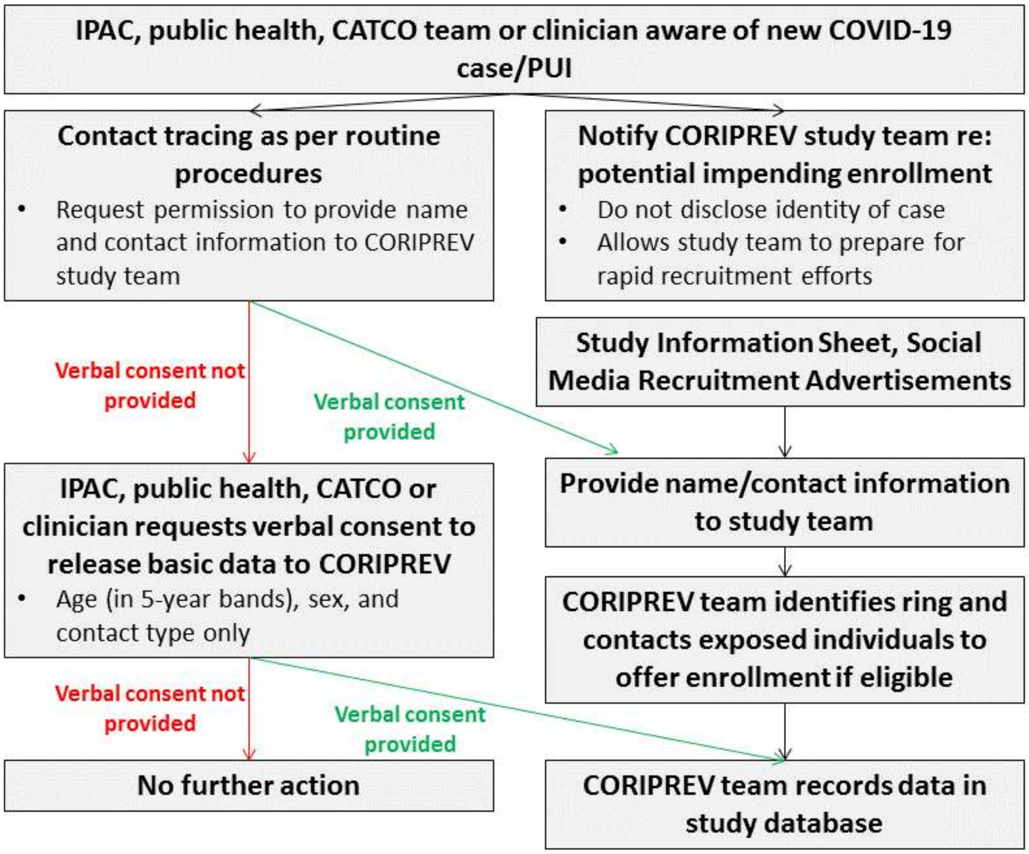

Second, the study team is also in regular contact with local public health units in both recruitment areas. As for the IPAC teams, in the event of a PUI/new case being identified, the public health team may alert the study team without disclosing any personal information about the case in order to help the study team prepare for rapid enrollment activities, and simultaneously conduct contact tracing as per routine procedures. In the course of conducting contact tracing, the public health team will ask exposed contacts for permission to provide their name and contact information to study staff; if provided, the study team will contact the individual to initiate recruitment activities.

Third, the study team includes the leadership of the CATCO trial, currently Canada's only clinical trial of COVID-19 treatment, being conducted among hospitalized patients (PI Murthy). For each participant enrolled into that trial, study staff will liaise with the relevant IPAC and/or public health team to again seek the verbal consent of exposed contacts to provide names and contact information to CORIPREV study staff, who will then contact the individuals to initiate recruitment.

Fourth, in Toronto, patients who test positive for COVID-19 at hospitals who belong to the Toronto Invasive Bacterial Disease Network (TIBDN) are being recruited into an REB-approved TIBDN surveillance, which permits approaching them about future

studies. Consenting patients in that TIBDN study who have household contacts will be asked if they would be willing to ask those household contacts for permission for staff from this CORIPREV trial to contact them about participation.

Fifth, front-line clinicians who are caring for index cases may become aware of our trial and wish to refer PUIs or new confirmed COVID-19 patients and their contacts. In such cases the clinician will be advised to speak with the index patient and will be asked to either a) contact their exposed close contacts and seek their consent to be contacted by the study team, before passing on this contact information on their behalf; b) pass on contact information for the study team to the exposed contacts; or c) otherwise arrange an introduction.

Finally, social media e.g. Facebook advertisements, will be employed to assist with recruitment. Advertisements will ask individuals exposed to a COVID-19 person and interested in research to contact the study team either through the CORIPREV email or by calling the St. Michael's main study coordinator. The study team will ensure identification of the ring(s) prior to offering eligible individuals enrollment into the study.

Because rapid initiation of PEP may be critical to its efficacy, recruitment of exposed individuals must occur as soon as possible after a confirmed COVID-19 case is identified. As such, a 48 hour time limit will be imposed to complete recruitment and enrollment activities, beginning at the time that the study team becomes aware of a confirmed diagnosis/index case. Those who cannot be contacted in time are ineligible for inclusion. Importantly, this 48 hour time limit refers strictly to the time window between the study team becoming aware of the index case and enrollment of exposed contacts. It does not refer to the time window after exposure between the case and contact. As per section 7.2, the study inclusion criteria allow participants to be enrolled if they were exposed to the index case within the past seven days. By also approaching the contacts of high-risk PUIs while awaiting confirmatory test results, the study team will allow more time for potential participants to be identified and for them to review the informed consent documents. To focus recruitment efforts where yield is highest, the team will prioritize PUIs where there is a strong pre-test probability of a positive result (eg. due to known contact with a confirmed case).

Individuals who decline to participate will be invited to complete a voluntary survey that collects basic demographic information, information on any symptoms they may have, as well as the reason for declining, This information will assist in recruitment strategies and help direct and gauge interest in potential future vaccine and treatment studies.

7.6 Enrollment Procedures

For this randomized trial, the ring (cluster of exposed individuals around each confirmed index case) is the unit of randomization, such that all enrolled members of the cluster will be assigned to the same study arm. Time to randomization and treatment may be critical to PEP efficacy; thus informed consent will occur in two stages to minimize

delays (section 16.2). In the first stage, all members of a ring who can be contacted within 48 hours will be asked to give preliminary consent by telephone or video link. This consent discuss should include all features required for obtaining full informed consent, as outlined in the 2nd edition of the Tri-Council Policy Statement on Ethical Conduct of Research Involving Humans (TCPS2),[84] and should be fully documented by the study coordinator. Those who provide verbal consent at this stage may be enrolled.

The second stage of the consent process will involve obtaining a signed copy of the REB-approved written informed consent form, either in electronic or hard copy. This stage should be done as soon as possible after the verbal preliminary consent is obtained, and the study staff must document that the participant has signed the consent form prior to any of the baseline study activities occurring, in accordance with Health Canada regulations. Hard copies of the consent form will be included in the participant kits to facilitate this step. Study staff should obtain a signed copy of the consent form no later than the day 35 visit, which is the first study visit that will routinely be done in-person in this trial (see sections 13.2-13.5 and section 16.2).

7.7 Co-enrollment Guidelines

Co-enrollment into other Health Canada-regulated clinical trials of COVID-19 prevention is permitted, but details of the co-intervention must be documented in the study database under the concomitant medication section or other suitable part of the database.

8. WITHDRAWAL OF PARTICIPANTS

8.1 Withdrawal criteria

Participants may be withdrawn from the study if the Sponsor-Investigator terminates the trial. There are no participant-specific criteria that necessitate withdrawal from the study. If a new health condition emerges in a participant randomized to the lopinavir/ritonavir arm that requires medications absolutely contraindicated by the protocol, then decisions regarding dose modification or discontinuation of the study drug can be made at the discretion of the site investigator, but the participant should be retained in the study for ascertainment of the primary and secondary outcomes. Reasons for the discontinuation of study drug should be noted in the study database.

9. RANDOMIZATION AND BLINDING PROCEDURES

9.1 Randomization

Each ring (cluster) will only be randomized once all members of a ring give preliminary consent, decline or are classified as not contactable within the 48 hour window for obtaining consent. It is essential to secure preliminary consent of the individual participants in a ring before randomization of the cluster in order to avoid selection bias, since this study is open-label.

Site study coordinators will use a secure web-based system that provides adequate allocation concealment to perform randomization, which will be in a 1:1 ratio with randomly permuted blocks of variable size and stratified by study site. This stratification is justified by the likely differences in transmission probability for these different exposure categories. The system will be housed at the Applied Health Research Centre at St. Michael's Hospital and randomization sequences pre-determined by a non-trial statistician. That statistician is the only person who will have access to the study codes. When interim analyses are conducted, only the codes related to the participants to be included in the interim analysis will be released to the biostatistician from the Data Safety and Monitoring Committee.

Each person who gives preliminary consent will be entered in the study database so it can provide an adequate number of uniquely coded study drug bottles for all consenting members of the cluster. An automated audit trail will record the time, date, allocation, and participant identification numbers. Each consenting participant will then have a baseline visit; those randomized to the active arm will receive study drug. Staff may travel to conduct visits for institutionalized participants. Those who withdraw preliminary consent or decline study drug will be asked to consent to be followed-up for the primary outcome. Only those who also decline follow-up will be excluded from intention-to-treat analyses.

9.2 Blinding

This trial is open-label at the level of the participant, study coordinator and investigator due to the impracticality of securing a supply of placebo. Lab technologists and statistical data analysts will be blinded to treatment allocation to minimize bias in the analysis.

10. STUDY INTERVENTIONS

10.1 Lopinavir/ritonavir

The study intervention is a 14-day course of lopinavir/ritonavir (LPV/r), to be initiated as soon as possible (but accepting up to seven days) after the last exposure. LPV/r is a commercially available product in Canada that was Health Canada approved for the treatment of HIV infection on March 9, 2001 under the trade name Kaletra®. An identical drug is marketed in other jurisdictions under the trade name Aluvia®, and differs only in the tablet coating (see section 10.1.3 below).

LPV/r is a fixed-dose combination product containing two anti-HIV drugs, which exert their antiviral effect through competitive inhibition of the HIV protease enzyme. Lopinavir was uniquely engineered to address shortcomings of older agents in the HIV protease inhibitor (PI) class (short plasma half-life, limited oral bioavailability, high degree of protein binding, susceptibility to common protease mutations). Ritonavir is an older agent in this same class. Lopinavir undergoes extensive first-pass metabolism through cytochrome P450 (CYP) enzymes 3A4 and 3A5, and coformulation with ritonavir exploits the latter's potent concentration-dependent inhibition of the CYP3A enzymes, thus decreasing hepatic metabolism of LPV and increasing plasma levels. While beneficial as a pharmacologic 'booster', ritonavir can also lead to clinically significant drug interactions. Ritonavir inhibits not only CYP3A but also 2D6, 2C9 and 2C19, although these effects are reduced significantly when coformulated with LPV.[85] Participants in this trial who are taking other medications and randomized to the LPV/r arm should undero assessment for drug-drug interactions by the site investigator. Consultation with a study pharmacist is available (see section 2, Study Contacts).

The 14 day duration is based on current estimates regarding the incubation period for COVID-19. Though estimates vary,[60] the mean is felt to be 6.4 days (95% credible interval=5.6-7.7; 97.5th percentile=11.1),[86] and the maximum expected incubation is similar to SARS and MERS at 14 days.[87-89]

10.1.1 Dosing and Administration

The dosage to be used in this study is 400/100 mg by mouth twice daily, or equivalent weight-based dosing (Table 1 below). LPV/r tablets can be taken with or without food. Tablets should not be chewed, broken or crushed. LPV/r oral solution should be taken with food or enteral nutrition when possible.

Of note, in the setting of HIV treatment, an alternative dosing strategy of 800/200 mg once daily has been evaluated and shown to be non-inferior to twice daily dosing for that indication.[90] However, because the pharmacodynamics for LPV/r's potential antiviral activity against SARS-CoV-2 are unknown, this trial will recommend twice daily dosing. The study team considered a loading dose of LPV/r at baseline (400/100 mg four times on day 1, followed by twice daily to complete 14 days total) in order to

optimize the pharmacokinetics of this drug against SARS-CoV-2. However this strategy was not selected due to concern that it may be associated with excessive side effects that could compromise further adherence with the study drug.

Table 1. Study drug dosing

Weight	Lopinavir dose taken twice daily	Volume (mL) of lopinavir/ ritonavir 80/20 mg/mL oral solution taken twice daily
7 to <15 kg<br>7 to 10 kg<br>>10 to <15 kg	12 mg/kg	 1.25 mL<br>1.75 mL
15 to 40 kg<br>15 to 20 kg<br>>20 to 25 kg<br>>25 to 30 kg<br>>30 to 35 kg<br>>35 to 40 kg	10 mg/kg	 2.25 mL<br>2.75 mL<br>3.50 mL<br>4.00 mL<br>4.75 mL
>40 kg	Use adult dosage of 400/100mg (2 x 200/50mg tablets) taken twice daily	5.00 mL (if unable to take tablets)

10.1.2 Dosing in special populations

LPV/r oral solution is contraindicated in pregnant women, participants with hepatic or renal failure and patients treated with disulfiram or metronidazole. LPV/r The oral solution contains the excipients alcohol (42.4% v/v) and propylene glycol (15.3% w/v). Avoid use of LPV/r oral solution in these population due to the alcohol content and potential propylene glycol toxicity.

10.1.3 Formulation and Packaging

LPV/r will be provided to the study team from the manufacturer Abbvie, using commercial supply. Because the global COVID-19 pandemic has placed significant demand for LPV/r, the study will use either Kaletra® or Aluvia®, depending on which product is available for immediate use. Both products are supplied in both tablet formulations whereas only Kaletra® has a liquid formulations; details of the chemical composition and appearance are provided below in Table 2. Study drug will be provided both in bottles of 56 tablets (200/50mg LPV/r per tablet) and bottles of 80 mL liquid (80/20 mg/mL concentration). Both study products will be labeled as

Investigational Product in accordance with Health Canada regulations.

Of note, Kaletra® is also available in 100/25 mg tablets in Canada (DIN 02312301) however this formulation will not be used in this study.

Table 2. Formulation, Appearance and Composition of Kaletra® and Aluvia®

	Kaletra®		Aluvia®
	**Tablet**	**Liquid**	**Tablet**
Formulation	200/50 mg	80/20 mg/mL	200/50 mg
DIN	02285533	02243644	N/A
Appearance	Yellow film-coated tablet	Light yellow / orange liquid	Red film-coated tablet
Non-medicinal ingredients	copovidone, colloidal silicon dioxide, sodium stearyl fumarate and sorbitan monolaurate	acesulfame potassium, alcohol, artificial cotton candy flavour, citric acid, glycerine, high fructose corn syrup, Magnasweet 110 flavour, menthol, natural and artificial vanilla flavour, peppermint oil, polyoxyl 40 hydrogenated castor oil, povidone, propylene glycol, saccharin sodium, sodium chloride, sodium citrate, water	Copovidone, Colloidal anhydrous silica, Sodium stearyl fumarate, Sorbitan laurate
Film-coating ingredients	polyethylene glycol 3350, polyvinyl alcohol, talc, titanium dioxide, yellow ferric oxide E172	N/A	Hypromellose<br>Titanium dioxide (E171)<br>Macrogols 400<br>Hydroxypropyl cellulose<br>Talc<br>Colloidal anhydrous silica<br>Macrogols 3350<br>Polysorbate 80<br>Iron Oxide Red (E172)

10.1.4 Storage and Stability

LPV/r film-coated tablets should be stored between 15 and 30°C. LPV/r oral solution should be stored between 2 and 8°C until dispensed. Avoid exposure to excessive heat. Keep cap tightly closed. Product must be stored and dispensed in the original container. Refrigeration of LPV/r oral solution by the participant is not required if used within 42 days and stored below 25°C.

10.1.5 Study Drug Dispensation

Study drug will be dispensed from the research pharmacy to study research coordinators, who will be responsible for arranging transportation to the study participants and documenting this process.

10.1.6 Expected Side Effects

LPV/r is considered a safe drug, but can be associated with gastrointestinal tolerability issues. Moderate/severe diarrhea, nausea and abdominal pain occurred in up to 28%, 16% and 11% respectively of adults taking LPV/r together with two other anti-HIV drugs in clinical trials.[52-55] Clinical trials using 28 days of LPV/r-based HIV PEP have shown GI side effects in ~half of participants, although discontinuations for adverse events were uncommon.[56-58]

Hyperlipidemia also occurs. In an observational cohort among 1278 treatment-naïve HIV-positive adults in France, changes in total cholesterol and triglycerides were +0.39 mmol/L and +0.40 mmol/L at one month.[91] However, such changes are asymptomatic, and would be expected to be transient with the 14 day regimen to be used in this trial. Routine measurement of serum lipids among people taking LPV/r for 28 days as HIV PEP was not recommended when this drug was the standard of care for that indication.

10.1.7 Additional Safety Considerations

The following safety considerations are based on data from HIV-positive individuals taking LPV/r in combination with other antiretroviral drugs after 48 weeks of daily use. They are listed here for reference but are not expected to be relevant to the short 14-day course of LPV/r used in this trial. In clinical trials and observational studies of HIV PEP, in which LPV/r was used in combination with other antiretroviral drugs for 28 days, none of the issues listed in this section were rated as frequent or severe.

- Pancreatitis should be considered if clinical symptoms (nausea, vomiting, abdominal pain) or abnormalities in laboratory values (such as increased serum lipase or amylase values) suggestive of pancreatitis should occur. Patients who exhibit these signs or symptoms should be evaluated and LPV/r should be

suspended as clinically appropriate. Pancreatitis has been observed in patients receiving LPV/r therapy, including those who developed marked triglyceride elevations. In some cases, fatalities have been observed. Although a causal relationship to LPV/r has not been established, marked triglyceride elevation is a risk factor for development of pancreatitis.

- Levels of blood glucose may increase. Such changes may in part be linked to the treatment per se, exacerbation of pre-existing diabetes mellitus, and hyperglycemia have been reported during post-marketing surveillance in HIV-1 infected receiving PI therapy.

- There have been reports of increased bleeding, including spontaneous skin hematomas and hemarthrosis, in patients with hemophilia type A and B treated with protease inhibitors (PIs).

- LPV/r is principally metabolized by the liver; therefore, caution should be exercised when administering this drug to patients with hepatic impairment, with monitoring of liver enzymes.

- Immune reconstitution inflammatory syndrome has been reported in patients with advanced HIV disease who are treated with combination antiretroviral therapy, including LPV/r. During the initial phase of treatment, patients responding to antiretroviral therapy may develop an inflammatory response to indolent or residual opportunistic infections [such as *Mycobacterium avium*-complex (MAC), cytomegalovirus (CMV), *Pneumocystis jirovecii pneumonia* (PCP), and tuberculosis (TB)], which may necessitate further evaluation and treatment.

- Autoimmune disorders (such as Graves' disease, polymyositis, Guillain-Barré syndrome, and autoimmune hepatitis) have also been reported to occur in the setting of immune reconstitution, however, the time to onset is more variable, and can occur many months after initiation of treatment.

- The presence of high level alcohol in LPV/r oral solution is potentially harmful for those suffering from liver disease, alcoholism, epilepsy, brain injury or disease, as well as for pregnant women and children. Patients taking LPV/r oral solution, particularly those with renal impairment or with decreased ability to metabolize propylene glycol (e.g., those of Asian origin), should be monitored for adverse reactions potentially related to propylene glycol toxicity (i.e., seizures, stupor, tachycardia, hyperosmolarity, lactic acidosis, renal toxicity, haemolysis).

- LPV/r oral solution contains up to 0.8 g of fructose per dose when taken according to the dosage recommendations. This may be unsuitable in hereditary fructose intolerance.

- LPV/r oral solution contains up to 0.3 g of glycerol per dose. Only at high inadvertent doses, it can cause headache and gastrointestinal upset.

Furthermore, polyoxol 40 hydrogenated castor oil and potassium present in LPV/r oral solution may cause only at high inadvertent doses gastrointestinal upset. Patients on a low potassium diet should be cautioned.

- Commonly reported adverse reactions to LPV/r during clinical trials included diarrhea, nausea, vomiting, hypertriglyceridemia and hypercholesterolemia. Diarrhea, nausea and vomiting may occur at the beginning of the treatment while hypertriglyceridemia and hypercholesterolemia may occur later.

- Dysgeusia (22%), vomiting (21%), and diarrhea (12%) were the most common adverse reactions of any severity and of probable, possible or unknown relationship to LPV/r oral solution in pediatric patients treated with combination therapy for up to 48 weeks.

10.2 Control arm

The comparator arm in this trial will be no intervention. This choice of control is justified on the basis of there being no known strategy for effectively preventing SARS-CoV-2 infection after exposure to a known case. A placebo comparator was considered, but found not to be feasible due to time constraints in sourcing a placebo and the urgency with which the study needs to be launched. The open-label design is anticipated not to bias the primary outcome of the trial, which is microbiologically confirmed evidence of SARS-CoV-2 infection on a respiratory specimen (section 6.4). Laboratory technicians and data analysts will be blinded to treatment allocation.

10.3 Concomitant and Prohibited Medications

Use of LPV/r together with medications cleared through cytochrome P450 3A (CYP 3A) can lead to elevated plasma concentrations of the concomitant medication. LPV/r also induces glucuronidation which may affect the exposure of some drugs. Medications that inhibit or induce CYP3A may increase or decrease serum concentrations of LPV/r, respectively. These drug-drug interactions may lead to adverse reactions, loss of therapeutic effect of the concomitant medication, and/or loss of antiviral activity of LPV/r.

Site investigators must therefore review concomitant medications at the time of enrollment. The medications listed in Table 3 are contraindicated, and use of any of these products that cannot be substituted during the study dosing period is an exclusion criterion for the study.

Table 3. Medications contraindicated for use with LPV/r

Drug Class	Drugs Within Class That Are Contraindicated with LPV/r	Clinical Comment
Alpha 1-adrenoreceptor antagonist	alfuzosin	Potential for serious reactions, such as hypotension.
Antiarrhythmic	dronedarone	Potential for cardiac arrhythmias.
Antibiotic	fusidic acid	Potential of increased fusidic acid-associated adverse events, such as hepatitis or bone marrow suppression.
Anticancer	apalutamide	Apalutamide is a moderate to strong CYP3A4 inducer and this may lead to a decreased exposure of LPV/r and potential loss of virologic response. In addition, exposure of apalutamaide may increase with co-administration of LPV/r that may lead to increased adverse events including seizure and fracture.
	neratinib	Potential for serious and/or life-threatening reactions including hepatotoxicity.
	venetoclax[b]	Concomitant use of strong CYP3A inhibitors, such as LPV/r, and venetoclax may increase the risk of tumor lysis syndrome at the dose initiation and during the ramp-up phase.
Antigout	colchicine, in patients with renal and/or hepatic impairment	Potential for serious and/or life-threatening reactions.
Antihistamines	astemizole[a], terfenadine[a]	Potential for serious and/or life-threatening reactions, such as cardiac arrhythmias.
Antimycobacterial	rifampin	Potential loss of virologic response and possible resistance to LPV/r or to the class of protease inhibitors or other co-administered antiretroviral agents. See Product Monograph for further details.
Antipsychotics	lurasidone	Potential for serious and/or life-threatening reactions.
	pimozide	Potential for serious and/or life threatening reactions, such as cardiac arrhythmias.
Ergot Derivatives	dihydroergotamine, ergonovine, ergotamine[a], methylergonovine[a]	Potential for serious and/or life-threatening reactions, such as acute ergot toxicity characterized by peripheral vasospasm and ischemia of the extremities and other tissues.
Gastrointestinal (GI) Motility Agent	cisapride[a]	Potential for serious and/or life-threatening reactions, such as cardiac arrhythmias.
Herbal Products	St. John's wort (Hypericum perforatum)	Potential loss of virologic response and possible resistance to LPV/r or to the class of

		protease inhibitors. See Product Monograph for further details.
Hepatitis C Virus (HCV) Direct Acting Antiviral	elbasvir/grazoprevir	Potential for the increased risk of alanine transaminase (ALT) elevations.
Lipid-modifying agents	lovastatin, simvastatin	Potential for serious reactions, such as risk of myopathy including rhabdomyolysis. See Product Monograph for further details.
	Lomitapide	Potential for serious reactions, such as hepatotoxicity.
Long Acting Beta-Adrenoceptor Agonist	salmeterol	Potential for increased risk of cardiovascular adverse events associated with salmeterol.
PDE5 Inhibitors	sildenafil[b], only when used for the treatment of pulmonary arterial hypertension (PAH)	Potential increase in PDE5 inhibitor associated adverse reactions including hypotension, syncope, visual changes and prolonged erection.
	Tadalafil, when used for the treatment of erectile dysfunction or PAH	Potential increase in PDE5 inhibitor associated adverse reactions including hypotension, syncope, visual changes and prolonged erection.
	vardenafil, when used for the treatment of erectile dysfunction or PAH	Potential increase in PDE5 inhibitor associated adverse reactions including hypotension, syncope, visual changes and prolonged erection.
Sedatives/Hypnotics	orally administered midazolam[b,c], triazolam	Potential for serious and/or life-threatening reactions, such as prolonged or increased sedation or respiratory depression.

[a] Product not marketed in Canada.
[b] See product monograph (Appendix X) for drug interaction recommendations.
[c] Oral formulation of midazolam is not marketed in Canada.

For other medications and/or supplements that are not on the list of contraindicated medications, site investigators must assess the risk of drug-drug interactions, and adjust medication doses accordingly. Two study pharmacists who have extensive experience with LPV/r use are available to provide advice to investigators regarding drug-drug interactions (see section 2, Study Contacts).

10.4 <u>Participant Access to Study Medication at Study Closure</u>

This study will provide access to 14 days of the study drug for those randomized to the intervention arm only. In the event that a study participant tests positive for COVID-19 during the follow-up period, efforts will be made to link the individual to ongoing clinical trials of COVID-19 treatment if such trials are enrolling in proximity to the study site.

However no guarantees of access to investigational product can be made. At the time of writing, there are no known definitively effective antiviral treatments for established SARS-CoV-2 infection.

11. RISK MANAGEMENT

11.1 Pregnancy

There are no eligibility restrictions regarding pregnancy in this study. However if a participant becomes pregnant during the study, the Investigator must inform the Sponsor-Investigator and collect follow-up data regarding the pregnancy, birth, and status of the child. Follow-up should be continued until study close-out at the study centre. Pregnancy is not an adverse event; however, any complication related to pregnancy should be considered an adverse event. Refer to Section 14.

There is considerable experience with the use of LPV/r in pregnancy among people living with HIV infection. In post-marketing surveillance through the Antiretroviral Pregnancy Registry, established since January 1989, based on prospective reports of over 3000 exposures to lopinavir containing regimens (including over 1000 exposed in the first trimester), there was no difference between lopinavir and overall birth defects compared with the background birth defect rate of 2.7% in the U.S. reference population of the Metropolitan Atlanta Congenital Defects Program.[51] Based on prospective reports from the APR of over 5000 exposures to ritonavir containing regimens (including over 2000 exposures in the first trimester) there was no difference between ritonavir and overall birth defects compared with the U.S. background rate.[51] For both lopinavir and ritonavir, sufficient numbers of first trimester exposures have been monitored to detect at least a 1.5 fold increase in risk of overall birth defects and a 2 fold increase in risk of birth defects in the cardiovascular and genitourinary systems. The prevalence of birth defects after any trimester exposure to LPV/r is comparable to the prevalence observed in the general population. The population exposed and monitored to date is only sufficient to detect major teratogenicity, and cannot detect an increase in the risk of relatively rare defects, however no pattern of birth defects suggestive of a common etiology was seen.

11.2 Breastfeeding

Studies in rats have demonstrated that lopinavir is secreted in milk, but it is not known whether it is secreted in human milk. Because of the potential for serious adverse reactions in nursing infants, mothers should be advised not to breastfeed while receiving LPV/r. Current breastfeeding is an exclusion criterion for this study.

11.3 Supportive Medications

Study sites may dispense supportive medication to participants in the active arm to help manage side effects of study drug. To minimize risk of exposure to and by participants (e.g. trips to the drug store) active arm participant kits may include over-the-counter dimenhydrinate 50mg (for management of nausea) and loperamide 2mg (for diarrhea).

Participants may be advised on dosing and frequency on an as needed basis by sites.

11.4 Protection of Study Personnel from COVID-19

COVID-19 is believed to be transmitted from person-to-person through respiratory droplets and via fomites. While the greatest risk of transmission is believed to occur in the context of respiratory symptoms, transmission from asymptomatic and minimally symptomatic individuals may occur. Participants in this trial will all have been recently exposed to a confirmed case of COVID-19 disease and will thus be at increased risk of having early or incubating infection during the 14 day incubation period. For these reasons, several measures will be taken to minimize the risk of transmission to study personnel.

First, the day 1, day 7 and day 14 study visits will generally be conducted remotely. If these study visits are more feasibly conducted in person (eg. in an institutionalized participant, in a healthcare worker already on site at the study site), then a surgical mask should be placed on the participant, and full droplet precaution personal protective equipment (PPE) should be worn by the staff during the study visit, including surgical mask, face shield or goggles, disposable gown, and gloves Prior to the day 35 in-person visit, study staff should ensure that the participant's prior oropharyngeal/nasal swabs are all SARS-CoV-2 negative, and that the participant is asymptomatic. The day 35 in-person visit can then be conducted using universal precautions (handwashing, gloves for phlebotomy) or whatever institution-specific precautions are recommended for patients not known to have COVID-19 in the context of the evolving epidemic. If a participant develops a positive oropharyngeal/nasal swab at any time point up to and including day 14, then the day 35 in-person visit should be delayed until all symptoms have resolved and at least 14 days have elapsed since the onset of the participant's symptoms, or until any institution-specific criteria for in-person visits in this scenario are met.

Any study staff person who develops symptoms potentially suggestive of COVID-19 disease should immediately be assessed by the site occupational health department and relieved of study duties until deemed safe to return to work.

It is possible that a study staff member or investigator may be identified as either an index COVID-19 case or an exposed contact in this study. In such cases there will be no restrictions on study eligibility imposed by the protocol, but it is advised the screening activities by handled by a site other than the site where the staff member is employed.

12. CLINICAL AND LABORATORY EVALUATIONS

12.1 Clinical Evaluations

Data collection procedures are modelled on the WHO FFX Master Protocol for COVID-19,[23-25] enabling comparability and potential pooling of study results with those in other jurisdictions. At baseline, study staff will conduct interviews to capture demographics; exposure assessment (relationship with case patient; timing/intensity/frequency of contact; protective measures taken during contact); medical history (comorbidities, medications, smoking) and symptoms. Follow-up interviews will be conducted at days 7, 14, 35 and 90. No physical examinations are required in this study, but participants will be asked to take their temperature daily from day 1-14. (See Section 9, Table 4: Schedule of Events)

Data will be recorded onto standardized electronic case report forms using the Research Electronic Data Capture system (REDCap) housed on secure servers at the Applied Health Research Centre (AHRC) at St. Michael's Hospital.

12.2 Laboratory Evaluations and Specimen Collection

12.2.1 HIV Self-testing

Participants over the age of 18 months in the active arm will be asked to undergo an HIV self-test using the INSTI HIV Self Test. The 18 month age limit is because antibody testing is not reliable in infacts under this age. This test is a single use, rapid, flow-through in vitro qualitative immunoassay for the detection of antibodies to Human Immunodeficiency Virus Type 1 (HIV-1) and Type 2 (HIV-2) in human fingerstick whole blood. The test is intended for use by untrained lay users as a self-test to aid in the diagnosis of HIV-1 and HIV-2 infection using one drop of blood obtained through a fingerstick collection procedure (Figure 3). For those who have difficulty performing the test, the test can be assisted by another person. For those under the age of 18 months, or for those in whom the test cannot feasibly be performed at baseline (eg. extreme fear of needles, limited manual dexterity), the test can be deferred until the day 35 visit at which time standard HIV testing should be done by the study coordinator (HIV RNA test in those aged <18 months; 4th generation antibody/antigen test for all others).

To perform the test, participants use a small lancet to obtain a drop of blood that is added to a small vial of test liquid. This first vial is then poured onto the test platform, followed by two other vials of liquid. The test device has a built-in control mechanism whereby a 'Control' dot is visible when the test is performed correctly. This 'Control' dot acts as the lay user's quality control tool. Appearance of the 'Control' dot indicates that the lay user has used correct amount and correct type of specimen for the test. The test result is considered as invalid if the 'Control' dot is not seen on the test device.

The INSTI HIV Self Test is a modification of its parent product, the INSTI HIV-1/HIV-2

Antibody Test for professional use, which is currently Health Canada approved (product number 90-1008, License 69580). The test principle and mechanism are identical for both INSTI HIV Self Test and INSTI HIV-1/HIV-2 Antibody Test. The INSTI HIV Self Test is currently CE marked, prequalified by the WHO, and approved for use in Kenya, Nigeria and Vietnam.

Figure 3. Excerpt from INSTI HIV Self-Test Instructions

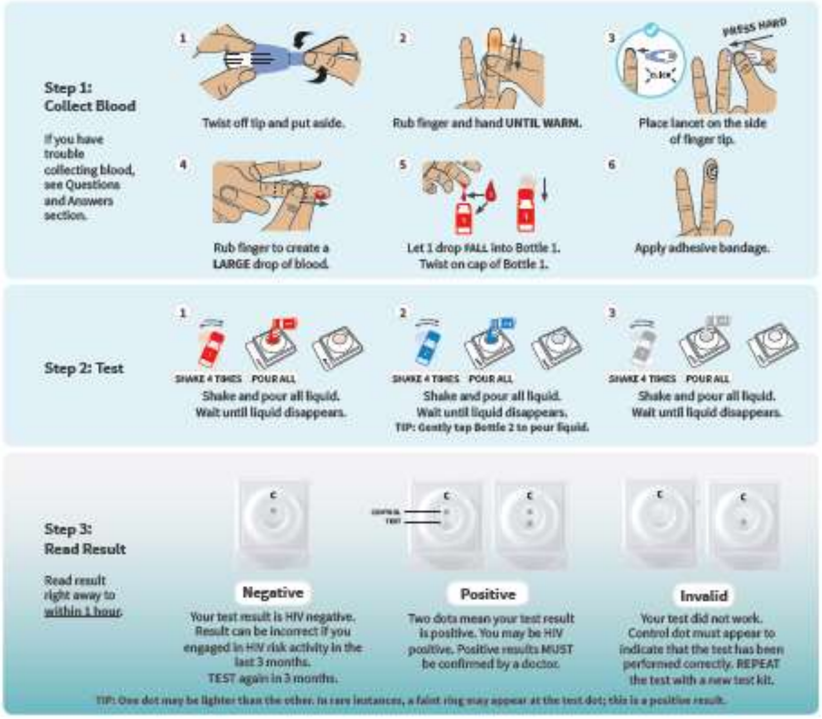

The performance and usability of the INSTI self test has been evaluated in three clinical studies with lay users in South Africa (HSTAR I and II), Kenya (KEMRI) and Congo Brazzaville. The study results of South Africa (HSTAR II) study have shown that 99.40% of the participants said the device was easy to use, 99.77% successfully completed entire testing process and 97.30% would recommend it to their sexual partner. Comparison of the Performance Efficacy Analysis of INSTI HIV Self Test from KEMRI, HSTAR III and Congo Brazzaville studies was excellent, with a sensitivity of 98.51-100% and specificity of 99.26-100%.[92]

12.2.2 Microbiologic testing for SARS-CoV-2

To obtain specimens for viral RNA testing, participants will be instructed on self-collection of oropharyngeal/nasal swabs as in prior work,[93,94] for RT-PCR testing.[95] Specimens will be collected at baseline, day 7, day 14 and within 24 hours of new symptoms. In participants who are asymptomatic, a second self-collected swab will also be collected at day 14 and combined in the same specimen vial, or a saliva specimen will be collected if swabs are not available, since having two respiratory specimens tends to increase sensitivity for the detection of respiratory viruses.[96,97] (If a participant has an earlier swab that is PCR positive then only one swab/saliva specimen needs to be collected at day 14.) NP swabs and deep respiratory samples (eg. bronchoalveolar

lavage/endotracheal tube aspirates) already done for clinical care purposes will also be collected, with biobanking of specimens not tested in real-time for future analysis. Specimens will be extracted using an automated system (eg. easyMAG) and real-time PCR conducted using primers and probes against genes encoding the RNA-dependent RNA polymerase (RdRp) and the envelope (E) protein of COVID-19. Quantitation against a standard curve will be calculated in RNA copies/microliter.

Participants not known to have COVID-19 at enrollment but whose baseline samples subsequently show SARS-CoV-2 will be permitted to continue in the trial and follow all study procedures, including completion of the 14-day course of study drug for those in the active arm, since such individuals offer an important opportunity to understand the role of the study drug in early treatment. The day 35 in-person visits should only be performed in-person if the participant becomes asymptomatic and if 14 days have elapsed since the onset of their symptoms, in order to minimize risks to study staff. Their data will be analyzed separately, as specified in section 15.3.1.

12.2.3 SARS-CoV-2 Serology

Methods for serologic testing of COVID-19 are still in development worldwide at the time of writing, but are rapidly emerging. Where possible (eg. in hospitalized participants, healthcare workers already at the healthcare facility, etc.), participants will undergo venipuncture at baseline, and samples processed into serum for frozen storage. Self-collected saliva samples at day 1, 7 and 14 may also be used for antibody detection. If a baseline sample cannot be collected, then for participants in the active arm, who will already be asked to obtain a drop of blood for HIV self-testing, a self-collected dried blood spot will be performed for this purpose. All participants will undergo venipuncture at day 35 for convalescent testing. Frozen samples will be batch-tested in the laboratory of Co-I Mubareka or other appropriate testing laboratory at the conclusion of the study when methods are finalized.

12.2.4 Oral microbiome analysis

Saliva samples will be collected at day 1, 7, 14 and 35 and these samples will be used for oral microbiome analysis to test whether specific taxa are associated with clinical outcome of COVID-19. DNA will be extracted from saliva samples and 16S rRNA sequencing will be done to identify microbiota composition pre-, during and post SARS-CoV-2 infection. Samples will be sent to lab of Dr. Gommerman at the University of Toronto.

12.2.5 Lopinavir levels

A random ~30% subset of participants in the active arm will have a self-collected dried blood spot drawn at day 14 for testing of plasma lopinavir levels as a biomarker of adherence and to assess for a pharmacokinetic correlate of protection.

12.3 Questionnaires

Questionnaires will be administered electronically through REDCap, which will be emailed to participants directly. Those lacking email/internet access may complete questionnaires on paper and/or by interview. For minors, the relevant guardian may discuss the questions and complete the questionnaires on their behalf if the participant is unable or prefers not to answer them independently.

Self-completed questionnaires will include a short set of demographic questions at baseline to characterize the sample. To maximize comparability across datasets, we will use standardized demographic questions from the Tri-Hospital/Toronto Public Health Health Equity Data Collection Research Project and questions from the Canadian Community Health Survey.[98]

All participants will be asked to complete self-reported online symptom and temperature diaries analogous to those used in prior influenza cohort studies.[64,99] The diaries will facilitate daily self-monitoring from days 1-14, (through questions asked on days 2-15 about the preceding day) followed by weekly monitoring until day 35 via a simple checklist that also grades the severity of symptoms. An email reminder will be sent for each questionnaire.

Questions about contacts who are known to develop symptoms and confirmed diagnoses will also be asked up to day 35, to assess for ongoing chains of transmission.

At day 14, participants will also be asked to complete the K10, a short ten-item scale to measure non-specific psychological distress. This rigorously developed instrument has differentiates between community cases and non-cases of mental health disorders according to the Structured Clinical Interview for DSM-IV (SCID).[76,77] A standard cutoff score of ≥16 will be used to define short-term psychological distress.

The psychological impact of COVID-19 exposure will be assessed at day 90 using the Impact of Event Scale (IES),[78] a well-characterized 15-item scale has also been used in studies of HCW exposed to pandemic H1N1 influenza.[100,101] This instrument, which includes a seven-item intrusion subscale and eight-item avoidance subscale, was designed to measure the longer term impacts of stressful life events and has good internal consistency, test-rest reliability and sensitivity to change.[78] A standard cutoff score of ≥26 will be used to define a traumatic stress response.[79] Additional questions related to practical and functional impacts of COVID-19 exposure and used in previous studies on the impact of the SARS epidemic will be included at this time point also.[79]

Multiple timepoints will incorporate the EQ-5D, a standardized instrument for assessing general health status and utility scores for incorporation into health economic analyses.[80] A utility score enables comparisons across different health interventions and diseases.[81]

12.4 Stored Research Specimens and Plans for Possible Future Testing

Any respiratory, serum and/or plasma specimens collected from participants but not analyzed in real-time will be stored in a biobank for potential future testing related to respiratory pathogen diagnostics and inflammatory biomarkers.

13. STUDY PROCEDURES

13.1 Schedule of Events

To protect study staff, efforts should be made to minimize unnecessary contact with participants during the 14 days after exposure to the index case. The baseline (day 1), day 7, day 14 and day 90 study visits are therefore designed to be conducted remotely whenever feasible, via telephone and/or video link in accordance with local regulations.

The baseline visit will occur as soon as possible (within 48 hours) after a contact is randomized ("day 1" regardless of the last date of contact with the active case). After baseline, data will be collected at days 7, 14, 35 and 90 (Table 4). In addition, using tools similar to those used in prior work on influenza,[64,99,102] participants will be asked to complete a daily online symptom diary from days 1-14, 21, 28 and day 35.[64,99] Adherence data will be captured using the same platform during the 14-day study drug period. Data collection from days 1-35 will permit detailed characterization of participants over the entire potential incubation period (section 10.1); the day 90 visit will capture later outcomes (psychological impact, hospitalization, mortality).

Table 4. Schedule of Events

Visit	Screening (Day 0)	Baseline (Day 1)	Day 7 ± 2d	Day 14 ± 2d	Day 35 ± 4d	Day 90 ± 2d
**Visit format**	**Remote**	**Remote**	**Remote**	**Remote**	**In person**	**Remote**
Eligibility assessment	X					
Informed consent/assent	X					
Cluster randomization	X[a]					
Dispensation of study drug (LPV/r arm)		X				
Interview by study staff		X	X	X	X	X
Concomitant medication assessment		X	X	X	X	X
Adverse event assessment			X	X	X	X
Adherence diary (LPV/r arm)		Daily on days 1-14				
Symptom diary		Daily on d2-15, then d21, d28, d35				
Temperature diary		Daily on d1-14				
Visit-specific questionnaire[b]		X		X	X	X
HIV self-test[c]		X				
Self-collected saliva sample		X	X	X	X	
Self-collected oropharyngeal/nasal swab[d]		X	X	X		
Self-collected dried blood spot		X[e]		X[f]		
Blood for SARS-CoV-2 serology		X[g]			X	
Stored blood samples					X	
Blood for CBC					X[f]	

[a] Randomization of the cluster occurs once all exposed contacts of an index COVID-19 case have provided their decision about study participation, or could not be contacted within 48 hours of the study team becoming aware of the index case; once randomization has been completed then site coordinators should contact individual participants to conduct the baseline activities.
[b] Specific components of each visit-specific questionnaire are listed in sections 13.3-13.7 below. Questionnaires can be completed on paper or via interview if internet access is unavailable. Guardians may complete questionnaires together with minors if preferred/not feasible by the minor.
[c] The HIV Self-test should only be done in participants over the age of 18 months; those under this age and those unable to perform the self-test for any reason (eg. extreme needle phobia, limited manual dexterity) should undergo standard HIV testing at day 35 (HIV RNA test in those aged <18 months; 4th generation antibody/antigen test for all others)
[d] An additional swab is to be collected within 24h if symptoms develop before day 14. Two respiratory specimens (swabs, saliva, etc.) are to be taken at day 14 timepoint, unless an extra

swab was already taken due to the development of symptoms in which case only one is collected.
NB: Other respiratory specimen(s) may be collected depending on availability of supplies
[e] The dried blood spot at baseline is to be performed in participants in the active arm only (since these participants are already collecting a drop of blood for the HIV self-test), for SARS-CoV-2 serology
[f] The dried blood spot at day 14 is to be performed in a random subsample of participants in the active arm only, for lopinavir levels. These participants are the only ones that require a CBC at day 35 since the hematocrit is required for interpretation of DBS lopinavir levels.[103,104]
[g] Baseline serology is to be collected at baseline only if the participant is institutionalized .

13.2 Screening Visit (Day 0; conducted remotely)

The purpose of this encounter is to rapidly assess participant eligibility and obtain preliminary consent to participate in the study, as a decision on participation is required from all members of a ring in order to randomize. The study coordinator should

- Interview the participant to assess eligibility criteria
- Provide and review the informed consent/assent document via email / fax / telephone / video link
- Obtain and document preliminary verbal consent/assent to participate; advise the participant that written consent/assent will be required as soon as possible.
- Obtain multiple forms of contact information for consenting/assenting participants, including multiple telephone numbers, an email address, and a mailing address for study material delivery.

13.3 Baseline Visit (Day 1; conducted remotely unless feasible in-person)

Once all members of a cluster have provided a decision regarding participation, or after 48 hours have elapsed since the study team first becomes aware of a confirmed case, the ring will be randomized and study coordinators should then re-contact consenting participants to initiate study activities. If the study visit is conducted remotely, an appropriate video-based platform should be used to facilitate instructions regarding oropharyngeal/nasal swab and, if indicated, HIV self-testing. If the study is conducted in person, appropriate PPE should be used. If an electronic or hard copy of the signed written informed consent form has not yet been obtained from the participant, this should be obtained as soon as possible, ideally at this visit. The participant must sign the consent form prior to the baseline study visit activities beginning. These activities include:

- **Dispense study drug:** For participants randomized to the active arm, study drug will be delivered to participants by courier (or in person if the study visit is conducted in person). The coordinator must confirm receipt of the package with the participant, and review dosing.

- **Interview:** The coordinator should obtain information from participants to complete the baseline case report forms, including medical history, exposure details, and concomitant medication assessment.
- **Diary/Temperature/Questionnaire instructions:** The coordinator should explain to the participant how to access and complete the online symptom/adherence diary (including temperature monitoring with the provided thermometer) and questionnaires, and encourage immediate completion of the baseline questionnaire on the day of the visit. Components at baseline include: demographics, EQ-5D, and HIV testing experience (active arm only)
- **HIV self-testing:** Participants randomized to the active arm are advised to self-administer a point-of-care HIV test at baseline, because use of the study drug LPV/r in those with undiagnosed HIV is suboptimal HIV therapy.[105,106] The study coordinator should perform routine pre-test counseling and guide the participant through the steps of self-testing as per the Operations Manual. Those found HIV-positive will be immediately linked to HIV care, through which they should undergo a confirmatory HIV serology test, and receive a fully active HIV regimen in addition to the study intervention as allocated; HIV positivity is not an exclusion criterion for the trial.
- **Self-collected dried blood spot:** Participants randomized to the active arm are asked to collect a dried blood spot
- **Self-collected saliva sample:** The participants are asked to collect saliva sample in a salivette tube. Participant should not eat, drink or brush their teeth for at least 30 minutes prior to collection. The coordinator should review the printed instruction on how to self-collect, label, date and package the salivette tube.
- **Self-collected oropharyngeal/nasal swab:** The coordinator should review the printed instructions on how to self-collect, label, date and package the oropharyngeal/nasal swab. The participant should then collect and date the sample and package it for pick-up.
- **Blood for serology (if visit conducted in person only):** The coordinator should collect one tube of blood for SARS-CoV-2 serology testing, process this sample into serum and store it at -80C.

13.4 Day 7 Visit (conducted remotely unless feasible in person)

This visit can be conducted remotely, via video link or telephone, or in person if feasible. If the study is conducted in person, appropriate PPE should be used. Study activities include:

- **Interview:** The coordinator should obtain information from participants to complete the day 7 case report forms, including symptoms, re-exposure details, concomitant medication assessment and adverse event assessment.
- **Diary/Questionnaire instructions:** The coordinator should remind the participant to complete the online daily symptom/adherence diary.
- **Self-collected saliva sample:** The participants are asked to collect saliva sample in a salivette tube. Participants should not eat, drink or brush their teeth

for at least 30 minutes prior to collection. The coordinator should review the printed instruction on how to self-collect, label, date and package the salivette tube.

- **Self-collected oropharyngeal/nasal swab:** The coordinator should review the printed instructions on how to self-collect, label, date and package the oropharyngeal/nasal swab. The participant should then collect and date the sample and package it for pick-up.

13.5 Day 14 Visit (conducted remotely unless feasible in person)

This visit can be conducted remotely, via video link or telephone, or in person if feasible. If the study is conducted in person, appropriate PPE should be used. Study activities include:

- **Interview:** The coordinator should obtain information from participants to complete the day 14 case report forms, including symptoms, re-exposure details, concomitant medication assessment and adverse event assessment.
- **Diary/Questionnaire instructions:** The coordinator should remind the participant to complete the online daily symptom/adherence diary and day 14 questionnaire. Components of the questionnaire at this visit include: EQ-5D, and the K10 psychological distress questionnaire. The coordinator should also remind the participant that they will receive a short day 21 and day 28 questionnaire which includes the symptom inventory
- **Self-collected saliva sample:** The participants are asked to collect saliva sample in a salivette tube. Participanst should not eat, drink or brush their teeth for at least 30 minutes prior to collection. The coordinator should review the printed instruction on how to self-collect, label, date and package the salivette tube.
- **Self-collected oropharyngeal/nasal swabs:** The coordinator should review the printed instructions on how to self-collect, date and package the two oropharyngeal/nasal swabs and place them in the same vial. The participant should then collect, label and date the sample.
- **Self-collected dried blood spot:** A 30% random subset of participants randomized to the active arm will be asked to collect a dried blood spot for lopinavir level testing

13.6 Day 35 Visit (conducted in person)

Universal precautions should be used for this visit.

If a paper (hard copy) of the signed written informed consent form has not yet been obtained from the participant, it must be obtained at this visit.

Prior to conducting this visit, study staff should ensure that the participant is asymptomatic and has had negative oropharyngeal/nasal swabs at previous time

points. If a participant has a oropharyngeal/nasal swab that is positive for SARS-CoV-2 at any prior timepoint, staff should conduct this visit in person only when at least 14 days have elapsed since the onset of the participant's symptoms, and the participant has been completely asymptomatic for at least 24 hours. Study activities include:

- **Interview:** The coordinator should obtain information from participants to complete the day 35 case report forms, including symptoms, re-exposure details, concomitant medication assessment and adverse event assessment.
- **Diary/Questionnaire instructions:** The coordinator should remind the participant to complete the day 35 questionnaire, which contains the symptom inventory, exposure history and EQ-5D.
- **Blood and saliva for antibody testing:** The coordinator should collect one tube of blood for SARS-CoV-2 serologic testing, process this sample into serum and store it at -80C. Saliva samples should be collected in a salivette tube and stored at -80C.
- **Stored blood samples:** The coordinator should collect one tube of blood each for processing into serum and plasma and storage at -80C. These samples will be used for lopinavir and ritonavir levels, and/or for measurement of inflammatory biomarkers.
  Blood for CBC: The 30% random subsample of active arm participants that collected a DBS for lopinavir levels will have a complete blood count drawn, because the hematocrit is required for interpretation of lopinavir levels

In the event that a participant cannot be contacted for the day 35 visit despite numerous attempts (at least 3), then the participant's alternative contacts should be contacted to ascertain whether the participant was hospitalized or became ill with COVID-19 disease.

13.7 Day 90 Visit (conducted remotely)

This visit should be conducted remotely, via video link or telephone. Study activities include:

- **Interview:** The coordinator should obtain information from participants to complete the day 90 case report forms, including symptoms, re-exposure details, concomitant medication assessment and adverse event assessment.
- **Questionnaire instructions:** The coordinator should remind the participant to complete the day 90 questionnaire, which contains the EQ-5D, IES, and related questions about impact of the COVID-19 exposure.

In the event that a participant cannot be contacted for the day 90 visit despite numerous attempts (at least 3), then the participant's alternative contacts should be contacted to ascertain whether the participant was hospitalized or became ill with COVID-19 disease.

13.8 Early Termination Visit

If a participant chooses to withdraw from the study prematurely after the baseline visit but before day 14, efforts should be made to complete all study activities required at the day 14 visit. Participants who discontinue study drug prematurely but consent to still be followed up in the study should follow routine protocol-defined procedures, particularly collection of the oropharyngeal/nasal swabs at the final visit.

If a participant chooses to withdraw from the study prematurely after the day 14 visit but before day 90, efforts should be made to complete all study activities required at the day 90 visit.

14. EVALUATION, RECORDING, AND REPORTING OF ADVERSE EVENTS

14.1 Definitions

14.1.1 Adverse Event (AE)

An AE is any untoward medical occurrence in a patient or clinical investigation participant, administered a study medication/intervention, which does not necessarily have a causal relationship with this treatment. An AE can therefore be any unfavorable and unintended sign (including an abnormal laboratory finding), symptom, or disease temporally associated with the use of a medicinal (investigational) study medication/intervention, whether or not related to the medicinal (investigational) study medication/intervention.

During each follow-up visit with the participant, information on AEs will be gathered and documented accordingly. AEs will be graded as mild, moderate, severe or life-threatening and assessed by causality as probably related, possibly related, unlikely to be related or not related to the study drug (investigational arm only).

Stable chronic conditions which are present prior to clinical trial entry and do not worsen are not considered AEs and will be accounted for in the participant's medical history.

14.1.2 Serious Adverse Events (SAEs)

An SAE is defined as an AE meeting one of the following criteria at any dose:

- Results in death during the period of protocol-defined surveillance
- Is a life-threatening event (defined as a participant at immediate risk of death at the time of the event)

- Results in in-patient hospitalization or prolongation of existing hospitalization during the period of protocol-defined surveillance
- Results in persistent or significant disability or incapacity (disability is defined as a substantial disruption of a person's ability to conduct normal life functions)
- Is a congenital anomaly or birth defect

Any other important medical event that may not result in one of the above outcomes, may be considered a SAE when, based upon appropriate medical judgment, the event may jeopardize the participant and may require medical or surgical intervention to prevent one of the outcomes listed above. Examples of such medical events include allergic bronchospasm requiring intensive treatment in an emergency room or at home, blood dyscrasias or convulsions that do not result in in-patient hospitalization, or the development of drug dependency or drug abuse.

Participants will be monitored during the 90-day study period for SAEs. If an SAE is ongoing at the time a participant discontinues/completes the study, the SAE will be followed until the Investigator agrees that the event is satisfactorily resolved, becomes chronic, or that no further follow-up is required.

14.2 AE Descriptions and Recording

Grading of AEs will be done by site investigators according to the Division of AIDS (DAIDS) Table for Grading the Severity of Adult and Pediatric Adverse Events

For all collected AEs (including SAEs), the site investigator will also determine the AE’s causality based on temporal relationship and his/her clinical judgment. The degree of certainty about causality will be graded using the categories below:

Definitely Related: There is clear evidence to suggest a causal relationship, and other possible contributing factors can be ruled out. The clinical event, including an abnormal laboratory test result, occurs in a plausible time relationship to Investigational Product administration and cannot be explained by concurrent disease or other products or chemicals. The response to withdrawal of the product (de-challenge) should be clinically plausible. The event must be pharmacologically or phenomenologically definitive, with use of a satisfactory re-challenge procedure if necessary.

Probably Related: There is evidence to suggest a causal relationship, and the influence of other factors is unlikely. The clinical event, including an abnormal laboratory test result, occurs within a reasonable time sequence to administration of the Investigational Product, is unlikely to be attributed to concurrent disease or other products or chemicals, and follows a clinically reasonable response on withdrawal (de-challenge). Re-challenge information is not required to fulfill this definition.

Possibly Related: There is some evidence to suggest a causal relationship (e.g., the event occurred within a reasonable time after administration of the trial medication). However, the influence of other factors may have contributed to the event (e.g., the participant's clinical condition, other concomitant events). Although an adverse drug event may rate only as "possibly related" soon after discovery, it can be flagged as requiring more information and later be upgraded to "probably related" or "definitely related", as appropriate.

Unlikely: A clinical event, including an abnormal laboratory test result, whose temporal relationship to Investigational Product administration makes a causal relationship improbable (e.g., the event did not occur within a reasonable time after administration of the trial medication) and in which other products or chemicals or underlying disease provides plausible explanations (e.g., the participant's clinical condition, other concomitant treatments).

Not related: The AE is completely independent of Investigational Product administration, and/or evidence exists that the event is definitely related to another etiology. There must be an alternative, definitive etiology documented by the clinician.

All SAEs which occur during the course of the study must be reported to the Project Manager based at St. Michael's Hospital within 24 hours of the site becoming aware of the event. The Project Manager will be responsible for reporting SAEs to Health Canada on behalf of the Sponsor-Investigator. SAEs will be reported to:

Project Manager:	Attia Qamar
Phone:	416-864-6060 x 77325
Fax:	416-864-5302
E-mail:	Attia.Qamar@unityhealth.to

14.3 Follow-up for Adverse Events

Any AE that occurs between the time that a study participant is randomized and the time that s/he departs the study at the end of the final study visit (or at the time of early discontinuation of the participant from the study for any reason) will be captured and recorded. At each contact with the participant, the investigator (or designate) must seek information on AEs by specific questioning and, as appropriate, by examination.

AEs that had previously been reported by the study participant will also be reassessed for duration, intensity and possible reoccurrence.

All AEs (including SAEs) will be followed until resolution or until the investigator and the clinical/medical monitor are in agreement that the AE has resolved, stabilized or become chronic and no further follow-up is required.

14.4 Pregnancy Follow-up

If a participant becomes pregnant during the study, the Investigator must inform the Sponsor-Investigator and collect follow-up data regarding the pregnancy, birth, and status of the child. Follow-up should be continued until study close-out at the study centre. Pregnancy is not an adverse event; however, any complication related to pregnancy should be considered an adverse event. Refer to Section 14.1.2.1 to 14.3.

15. STATISTICAL CONSIDERATIONS

15.1 General Study Design

This is a cluster-randomized, open-label trial using an adaptive design to sample size estimation.

15.2 Sample Size Considerations/Justification

This trial will use an adaptive approach to sample size calculation, in that the un-blinded DSMC statistician will examine rates of the primary outcome, ICC and the average and variance of the cluster sizes at the interim analysis; these results will be used to adjust the sample size in accordance with adaptive design principles.[72,73]

The preliminary sample size calculation is based on transmission parameters from historic literature on prior respiratory viruses and emerging data on COVID-19, with assumptions about heterogeneity in transmission that is intrinsic to outbreaks of emerging infections. Data from the 2003 experience with SARS in Beijing show a secondary attack rate (p_0; proportion of those exposed who get infected) of 6.3% (95%CI 5.3-7.3%), ranging from 0.36% (0-0.77%) in work/school contacts to 10.0% (0.7-19.3%) in friends and 15.4% (11.5-19.2%) in spouses.[107] Data from Singapore[108] and for HCW in Toronto during SARS was similar at 18.2%.[109,110] For seasonal and pandemic influenza household contacts, p_0=10-20%.[111-114] A recent report suggests a p_0 as high as 35% (95%CI=27-44) for COVID-19. As our initial estimate, we conservatively assume that p_0=15%. The number of contacts per case may vary widely (mean=3.8, range 1-80 for SARS in Beijing[107]); in estimating our initial target sample size, we considered values ranging from 5-20 in intervals of 5. We consider intra-class correlation coefficient (ICC) values of 0.02 and 0.05.[69]

To detect a relative risk reduction of 40% (the effect size observed in the MERS-CoV LPV/r PEP study[18]) with 80% power at a two-sided α=0.05 after accounting for one interim analysis according to Haybittle-Peto (see below), from 15% in the control group (p_0) to 9% in the intervention group, an average of 5 contacts per case and ICC=0.05, we require 110 clusters per arm, or 220 clusters overall. These estimates are consistent

with simulations assuming numbers of secondary infections per index case follow a negative binomial distribution.[115,116] To conservatively account for up to 10% loss-to-follow-up, the target has been inflated to **244 clusters total**, or ~1220 exposed people.

Importantly, p_0 and ICC are unknown for COVID-19, but are crucial drivers of statistical power.[72,117] As noted above, the trial will thus incorporate an adaptive trial design[72,73,118] to improve the sample size calculations at the time of the interim analysis, while keeping investigators blinded to treatment allocation and effect.

15.3 Statistical Analyses

15.3.1 Analysis of Primary Outcome Measures

The primary outcome is microbiologically confirmed SARS-CoV-2 infection, ie. detection of viral RNA in a respiratory specimen (oropharyngeal/nasal swab, nasopharyngeal swab, sputum specimen, saliva specimen, oral swab, endotracheal aspirate, bronchoalveolar lavage specimen) by day 14. Our primary analysis will be a generalized linear mixed model (GLMM) with logit link to estimate the effect of LPV/r on the probability of infection while accounting for clustering of participants in rings, with stratification by the randomizing site. Multivariable models will adjust for key characteristics of both contacts (age, sex, co-morbidity, exposure characteristics such as type, duration and timing) and index cases (illness severity, concomitant medications etc.). In particular, we will include sex/gender-based analyses with variables specific to contacts and types of exposures.

Participants with specimens positive for SARS-CoV-2 at baseline will be permitted to continue in the trial and follow all study procedures, including completion of the 14-day course of study drug for those in the active arm, as noted in section 12.2.2. These participants will be analyzed separately from all other participants, as these analyses will provide exploratory data regarding early LPV/r treatment rather than PEP.

15.3.2 Analysis of Secondary Outcome Measures

To assess safety, adverse events will be tabulated according to grade and causality assessment using definitions from the DAIDS Table for Grading the Severity of Adverse Events,[75] and compared between study arms.

As for the primary analysis outlined in section 15.3.1 above, GLMM with logit link (for dichotomous outcomes) or identity link (for continuous outcomes) will also be used to compare the following secondary outcomes between study arm:

- symptomatic COVID-19 disease by day 14
- seropositivity by day 35
- hospitalization attributable to COVID-19 disease by day 90

- respiratory failure attributable to COVID-19 disease requiring non-invasive ventilation by day 90
- mortality attributable to COVID-19 disease
- short-term psychological distress, defined as scoring ≥16 on the K10[76,77] at day 14
- long-term traumatic stress response, defined as scoring ≥26 on the IES scale[78,79] at day 90
- health-related quality of life, measured by the EQ-5D-5L on days 1, 14, 35 and 90

We will also consider comparing the onset of symptomatic disease, seroconversion, hospitalization, respiratory failure and mortality in time-to-event models. All analyses will be by intention-to-treat. While loss to follow-up is expected to be low, missing data will be imputed under a variety of assumptions, to determine the robustness of our findings.

For all secondary analyses, participants with specimens positive for SARS-CoV-2 at baseline will be analyzed separately from all other participants.

15.3.3 Descriptive analyses

Participant and cluster characteristics will be summarized using descriptive statistics. These analyses will characterize exposure histories, numbers of contacts per index case, and feasibility of rapidly contacting exposed contacts. Data from the control arm only will be used to characterize the transmission-related epidemiologic parameters and natural history of COVID-19 among exposed contacts in the Canadian context, including estimation of the secondary attack rate, viral shedding (time to first shedding, duration, cycle threshold), and symptom burden. We will use multivariable models to examine factors associated with cluster size, as well as incident infection, cycle threshold and symptom development, considering the index case as the clustering variable as appropriate, and considering factors related to both the index case (age, sex) and the secondary contacts (demographics, timing of contact, etc.).

15.4 Subgroup Analyses

Subgroup analyses of the primary outcome will be performed according to the type of exposed contact (eg. elderly in congregate living facilities, HCW, community) since prior data have suggested considerably different secondary attack rates for respiratory viruses in these settings, and according to site/province, since small differences related to practice patterns and other unknown factors may exist. Subgroup analyses will also be performed according to the symptom status of the index case (symptomatic versus asymptomatic at the time of contact).

15.5 Interim Analyses

An interim analysis will be conducted when 122 clusters have been followed-up for the primary outcome, adjusting the significance level according to Haybittle and Peto.[119] The DSMC will make recommendations at this point regarding whether to continue the trial as is, or stop the trial for futility, with consideration of studying an alternative promising agent as COVID-19 PEP or otherwise altering the study intervention as applicable (see section 17.2.1).

16. ETHICAL CONSIDERATIONS

16.1 Ethical Conduct of the Study

This study will be conducted in accordance with the ICH-GCP Guidelines and the principles in the Declaration of Helsinki. Investigators will be thoroughly familiar with the appropriate use of the study drug as described in the protocol and product monograph.

16.2 Informed Consent/Assent

Because of the cluster randomized design of this trial, consent will be obtained in two stages, as outlined also in sections 13.2-13.5. The first stage of consent will be obtained when first contacted by study staff. At this point participants will be given detailed oral information about the study; wherever possible (ie. if the individual agrees to correspond immediately via email or fax), detailed written information about the study should also be provided at this time by sending the informed consent form. Each participant should have sufficient opportunity to discuss the study, have all of their questions addressed and consider the information in the consent process prior to agreeing to participate. If at least verbal consent/assent is obtained at this time, this consent/assent and the consent/assent process must be documented in writing by the study coordinator in accordance with principles outlined in the 2nd edition of the Tri-Council Policy Statement on Ethical Conduct of Research Involving Humans (TCPS2).[84] The study staff must document that the participant has signed the consent form prior to any of the baseline study activities occurring, and hard copies of the consent form will be included in the participant kits to facilitate this step. Study staff should obtain a signed copy of the consent form no later than the day 35 visit, which is the first study visit that will routinely be done in-person in this trial. The screening visit activities can then occur. Baseline activities cannot occur until after randomization of the cluster, which requires this preliminary consent/assent.

The second stage of the consent/assent process will involve obtaining a signed copy of the REB-approved written informed consent/assent form, either in electronic or hard copy. This stage should be done as soon as possible after the verbal preliminary

consent is obtained, and must be completed by the day 35 visit, which is the first visit routinely conducted in person.

Participants may withdraw consent at any time during the course of the study without prejudice. The informed consent form will be signed and dated by the participant and the investigator or delegate. The original signed informed consent form will be retained in the participant's study files for 25 years and a copy will be provided to the participant.

16.3 Confidentiality

All participant-related information including Case Report Forms, laboratory specimens, evaluation forms, reports, etc. will be kept strictly confidential. All records will be kept in a secure, locked location and only accessible to research staff. Participants will be identified only by means of a coded number specific to each participant. All computerized databases will identify participants by numeric codes only, and will be password protected.

Upon request, and in the presence of the investigator or his/her representative, participant records will be made available to the study Sponsor-Investigator, monitoring groups representative of the study Sponsor-Investigator, representatives of funding groups, and applicable regulatory agencies for the purpose of verification of clinical trial procedures and/or data, as is permissible by local regulations.

16.4 Institutional Review Board, Ethics Committee, or Research Ethics Board

The REB will review all appropriate study documentation to safeguard the rights, safety, and well-being of the participants. The study will be conducted only at sites where ethics approval has been obtained. A copy of the protocol (including protocol amendments), all versions of informed consent forms, other information to be completed by participants such as survey instruments or questionnaires, and any proposed recruitment materials must be reviewed and approved by the REB of each participating centre prior to implementation of the trial. The investigator will be responsible for obtaining REB approval of the annual Continuing Review throughout the duration of the study. The investigator will notify the REB of serious adverse events as applicable. The investigator will seek prior ethics approval for any protocol deviations except when the change is intended to eliminate an immediate hazard to participants. In this case, the protocol deviation will be promptly reported.

17. General Trial Conduct Considerations

17.1 Adherence to Protocol

17.1.1 Protocol Amendments

All protocol amendments will be reviewed and approved and if applicable submitted to the applicable regulatory agencies for prior approval or notification. The Investigator must sign and date the amendment prior to implementation. All protocol amendments must also be submitted to the ethics committee.

17.1.2 Protocol Deviations

No deviations from this protocol will be permitted without the prior written approval of the Sponsor-Investigator, except when the modification is needed to eliminate an immediate hazard or hazards to participants. Any deviations that may affect a participant's treatment or informed consent, especially those increasing potential risks, must receive prior approval from the REB unless performed to remove an immediate safety risk to the participants. In this case it will be reported to the REB and the Sponsor-Investigator immediately thereafter. Any departures from the protocol must be documented.

17.2 Monitoring & Auditing

17.2.1 Data Safety Monitoring Committee

The DSMC will include infectious diseases and public health specialists, at least one biostatistician, and researchers with an understanding of emerging infections and clinical trials. The DSMC will meet at the time of the interim analysis and bi-annually. The purpose of the DSMC will be to review safety concerns, advise on revisions to the sample size in accordance with the adaptive trial design (section 15.2), conduct and review the interim analysis (section 15.5), and review external data that may have bearing on the design of or decision to continue the trial.

In the event that the outbreak has ended at one or more study sites and the study has not yet been completed due to insufficient enrollment, the study should be paused. Efficacy data from the trial must not be prematurely released. In this instance, the DSMC should conduct an interim analysis of the study data to make recommendations whether the study should continue or stop for efficacy, futility or safety. As per World Health Organization policies on data-sharing in a Public Health Emergency, clinical trial outcome data will be shared at the earliest possible opportunity.

17.2.2 Study Monitoring

Each study site agrees to allow representatives of the trial data management centre (Applied Health Research Centre of St. Michael's Hospital; AHRC) to have direct access to the study records and medical records from those patients enrolled in the clinical study as well as Investigational Product accountability records, in order to conduct remote risk-based monitoring of this trial. The proposed remote monitoring scheme will be composed of:

1) Centralized review of essential study document at all participating sites related to participant protection, such as ICF signature pages, GCP and protocol training records, Delegation log, CV and medical license of investigators and Protocol signature page
2) Targeted source data verification (SDV) of eCRF data will be performed on 10% of participants chosen at random. Only critical data variables identified through risk assessment which are programmed into the EDC system will be source verified.

AHRC will provide sites with the SDV Tool, which is a one page document listing key variables for targeted SDV. Sites will be asked to complete the SDV Tool and send it to the central coordination team, along with de-identified source documents for selected variables listed on the SDV Tool via a dedicated end-to-end secure internet portal. Instructions will be provided to sites on how to complete the SDV tool, de-identify source documents, and send these documents to central coordination team using the secure portal.

Upon receipt of the completed SDV Tool and appropriate source documents, the central coordination team will review these documents to ensure that the data is consistent with the data entered on eCRF. The review will include checking the eCRF entries for accuracy and completeness against source documents. AHRC will maintain a monitoring log of all patients for whom source documents are requested, received, and verified. Variables will be marked as "verified" in the monitoring log once the review is complete. Urgent issues will be communicated on an ongoing basis as needed with the PIs/Sponsor.

If errors or inconsistencies are noted, a follow up email will be sent to the site's Principal Investigator and Primary Study Coordinator. The follow up email will include a summary of the issues identified, outline of any corrective actions and/or request an explanation, and a timeline for resolution.

Continuous, binary and categorical data will be examined for evidence of fraud at the centre level with simple graphical representations and statistical tests. If data appears to have some signs of potential fabrication, further analysis will be done by looking at digit preference for continuous variables. Data with be visually examined with a histogram and preference will be compared with a two by two chi-squared test. In addition, compliance with Benford's Law can be considered.

17.3 Record Keeping

17.3.1 Data Collection

The Investigator must maintain detailed records on all study participants. Data for this study will be recorded in the participant's chart and entered into CRFs. Applicable data from the participant's chart should be recorded in the CRFs completely and promptly, taking time to correct any mistakes. Copies of CRFs will remain at the clinical site at the conclusion of the study.

17.3.2 Source Documents

The Investigator must maintain adequate and accurate source documents upon which CRFs for each participant are based. They are to be separate and distinct from CRFs except for cases in which the Sponsor-Investigator has pre-determined that direct data entry into specified pages of the participant's CRF is appropriate. These records should include detailed notes on:

- Oral and written communication with participant regarding the study treatment (risks/benefits)
- Participation in trial and signed and dated informed consent forms
- Inclusion and exclusion criteria details
- Visit dates
- Adverse events and concomitant medication
- Laboratory result printouts
- Participant's exposure to any concomitant therapy
- Reason for premature discontinuation (if applicable)
- Enrollment number
- Adherence with the study protocol and protocol deviation information

17.3.3 Data Corrections

Any corrections of data entered on source documents at the study site should be crossed out with a single horizontal line, initialed and dated.

17.3.4 Data Management

Data management responsibilities for this trial will be assumed by the AHRC. Instructions concerning the recording of study data on CRFs will be provided in a comprehensive study Operations Manual. Each study site is responsible for submitting the data in a timely fashion. Detailed aspects of data handling will be described in the Data Management Plan.

17.3.5 Record Retention

The Investigator will maintain all study records according to the ICH-GCP and applicable Health Canada regulatory requirements. Records will be retained for 25 years, in accordance with applicable Health Canada regulatory requirements. If the Investigator withdraws from the responsibility of keeping the study records, custody must be transferred to a person willing to accept the responsibility and the Sponsor-Investigator notified. The Investigator should ensure that no destruction of medical records occurs without the written approval of the Sponsor-Investigator.

17.4 Steering Committee

The steering committee (SC) will include the three Co-Principal Investigators (Tan, McGeer, Chan), together with other pre-selected investigators with appropriate methodologic and content expertise. The SC will meet 1-2 monthly to discuss epidemic trends/recruitment, consider new sites, ethical issues, and emerging scientific or logistical issues. The SC will advise the Co-PIs on relevant trial decisions, but final decision-making authority will rest with the Co-Principal Investigators.

18. Disclosure and Publication Policy

18.1 Publication of Study Results

Following completion of the study, the lead Principal Investigator is expected to publish the results of the primary and secondary analyses from this trial, as well as the health economic analyses, in peer-reviewed scientific journals. A detailed authorship policy will be developed and agreed upon by all investigators to determine how best to fairly acknowledge the contributions of relevant parties.

18.2 Data Sharing for Secondary Research

Data from this study may be used for secondary research. All of the individual participant data collected during the trial will be made available after de-identification through expert determination. These data will be made available as soon as possible following publication, with no end date, as part of data sharing requirements from journals and funding agencies, and in the spirit of open data access.

18.3 Conflict of interest

The independence of this study from any actual or perceived influence, such as by the pharmaceutical industry, is critical. Therefore, any actual conflict of interest of persons who have a role in the design, conduct, analysis, publication, or any aspect of this trial will be disclosed and managed. Furthermore, persons who have a perceived conflict of interest will be required to have such conflicts managed in a way that is appropriate to their participation in the design and conduct of this trial.

19. REFERENCES

1. Wang D, Hu B, Hu C, et al. Clinical Characteristics of 138 Hospitalized Patients With 2019 Novel Coronavirus-Infected Pneumonia in Wuhan, China. Jama 2020;7.
2. Chen N, Zhou M, Dong X, et al. Epidemiological and clinical characteristics of 99 cases of 2019 novel coronavirus pneumonia in Wuhan, China: a descriptive study. Lancet 2020;30:30211-7.
3. Huang C, Wang Y, Li X, et al. Clinical features of patients infected with 2019 novel coronavirus in Wuhan, China. Lancet 2020;24:30183-5.
4. Chan JF, Yuan S, Kok KH, et al. A familial cluster of pneumonia associated with the 2019 novel coronavirus indicating person-to-person transmission: a study of a family cluster. Lancet 2020;24:30154-9.
5. Riou J, Althaus CL. Pattern of early human-to-human transmission of Wuhan 2019 novel coronavirus (2019-nCoV), December 2019 to January 2020. Euro Surveill 2020;25(4).10.2807/1560-7917.ES.2020.25.4.2000058.
6. Nasser S, Blum B. Canada's 1st 'presumptive' case of coronavirus found in Toronto. https://www.cbc.ca/news/canada/toronto/canada-1st-case-coronavirus-toronto-1.54407602020.
7. Silverstein WK, Stroud L, Cleghorn GE, Lei JA. First imported case of 2019 novel coronavirus in Canada, presenting as mild pneumonia. Lancet 2020.
8. Althaus CL. Estimating case fatality ratio of COVID-19 from observed cases outside China. https://github.com/calthaus/ncov-cfr. Accessed Feb 18, 2020. 2020.
9. Polack FP, Thomas SJ, Kitchin N, et al. Safety and Efficacy of the BNT162b2 mRNA Covid-19 Vaccine. N Engl J Med 2020;383:2603-15.
10. Baden LR, El Sahly HM, Essink B, et al. Efficacy and Safety of the mRNA-1273 SARS-CoV-2 Vaccine. N Engl J Med 2021;384:403-16.
11. Kim JH, Marks F, Clemens JD. Looking beyond COVID-19 vaccine phase 3 trials. Nat Med 2021.
12. Attwell K, Lake J, Sneddon J, Gerrans P, Blyth C, Lee J. Converting the maybes: Crucial for a successful COVID-19 vaccination strategy. PLoS One 2021;16:e0245907.
13. Tegally H, Wilkinson E, Lessells RJ, et al. Sixteen novel lineages of SARS-CoV-2 in South Africa. Nat Med 2021.
14. Phan LT, Nguyen TV, Luong QC, et al. Importation and Human-to-Human Transmission of a Novel Coronavirus in Vietnam. N Engl J Med 2020;28.
15. Li Q, Guan X, Wu P, et al. Early Transmission Dynamics in Wuhan, China, of Novel Coronavirus-Infected Pneumonia. N Engl J Med 2020;29.
16. Nishiura H, Kobayashi T, Yang Y, et al. The Rate of Underascertainment of Novel Coronavirus (2019-nCoV) Infection: Estimation Using Japanese Passengers Data on Evacuation Flights. J Clin Med 2020;9(2).jcm9020419. doi: 10.3390/jcm.
17. Chang D, Xu H, Rebaza A, Sharma L, Dela Cruz CS. Protecting health-care workers from subclinical coronavirus infection. Lancet Respir Med 2020.
18. Park SY, Lee JS, Son JS, et al. Post-exposure prophylaxis for Middle East respiratory syndrome in healthcare workers. J Hosp Infect 2019;101:42-6. doi: 10.1016/j.jhin.2018.09.005. Epub Sep 18.
19. Tan D, Walmsley S. Lopinavir plus ritonavir: a novel protease inhibitor combination for HIV infections. Expert Rev Anti Infect Ther 2007;5:13-28. doi: 10.1586/14787210.5.1.13.

20. Panel on Antiretroviral Guidelines for Adults and Adolescents. Guidelines for the Use of Antiretroviral Agents in Adults and Adolescents with HIV. Department of Health and Human Services. Available at http://www.aidsinfo.nih.gov/ContentFiles/AdultandAdolescentGL.pdf. Accessed Feb 17, 2020. 2020.
21. Ford N, Shubber Z, Calmy A, et al. Choice of antiretroviral drugs for postexposure prophylaxis for adults and adolescents: a systematic review. Clin Infect Dis 2015;60:S170-6. doi: 10.1093/cid/civ092.
22. Wang M, Wu Q, Xu W, et al. Clinical diagnosis of 8274 samples with 2019-novel coronavirus in Wuhan. https://www.medrxiv.org/content/10.1101/2020.02.12.20022327v1.full.pdf Accessed Feb 18, 2020. medRxiv 2020.
23. The First Few X (FFX) Cases and contact investigation protocol for 2019-novel coronavirus (2019-nCoV) infection. Version 2. https://www.who.int/publications-detail/the-first-few-x-%28ffx%29-cases-and-contact-investigation-protocol-for-2019-novel-coronavirus-%282019-ncov%29-infection Accessed Feb 18, 2020. World Health Organization.; 2020.
24. Protocol for assessment of potential risk factors for 2019-novel coronavirus (2019-nCoV) infection among health care workers in a health care setting. https://www.who.int/publications-detail/protocol-for-assessment-of-potential-risk-factors-for-2019-novel-coronavirus-%282019-ncov%29-infection-among-health-care-workers-in-a-health-care-setting Accessed Feb 18, 2020. World Health Organization; 2020.
25. Household transmission investigation protocol for 2019-novel coronavirus (2019-nCoV) infection. https://www.who.int/publications-detail/household-transmission-investigation-protocol-for-2019-novel-coronavirus-%282019-ncov%29-infection Accessed Feb 18, 2020. World Health Organization; 2020.
26. Zhou P, Yang XL, Wang XG, et al. A pneumonia outbreak associated with a new coronavirus of probable bat origin. Nature 2020;3:020-2012.
27. Zhang T, Wu Q, Zhang Z. Pangolin homology associated with 2019-nCoV. bioRxiv 2020:2020.02.19.950253.
28. Wong MC, Javornik Cregeen SJ, Ajami NJ, Petrosino JF. Evidence of recombination in coronaviruses implicating pangolin origins of nCoV-2019. bioRxiv 2020:2020.02.07.939207.
29. World Health Organization. COVID-19 Situation Report-36. https://www.who.int/docs/default-source/coronaviruse/situation-reports/20200225-sitrep-36-covid-19.pdf?sfvrsn=2791b4e0_2.
30. World Health Organization. COVID-19 Situation Report-42. Geneva: Switzerland. Available at: https://www.who.int/docs/default-source/coronaviruse/20200302-sitrep-42-covid-19.pdf?sfvrsn=d863e045_2.
31. Lu H. Drug treatment options for the 2019-new coronavirus (2019-nCoV). Biosci Trends 2020;28:01020.
32. Sheahan TP, Sims AC, Graham RL, et al. Broad-spectrum antiviral GS-5734 inhibits both epidemic and zoonotic coronaviruses. Sci Transl Med 2017;9(396).9/396/eaal3653. doi: 10.1126/scitranslmed.aal3653.

33. de Wit E, Feldmann F, Cronin J, et al. Prophylactic and therapeutic remdesivir (GS-5734) treatment in the rhesus macaque model of MERS-CoV infection. Proc Natl Acad Sci U S A 2020;13:1922083117.
34. Wang M, Cao R, Zhang L, et al. Remdesivir and chloroquine effectively inhibit the recently emerged novel coronavirus (2019-nCoV) in vitro. Cell Res 2020;30:269-71. doi: 10.1038/s41422-020-0282-0. Epub 2020 Feb 4.
35. Jo S, Kim H, Kim S, Shin DH, Kim MS. Characteristics of flavonoids as potent MERS-CoV 3C-like protease inhibitors. Chem Biol Drug Des 2019;94:2023-30. doi: 10.1111/cbdd.13604. Epub 2019 Sep 12.
36. Li G, De Clercq E. Therapeutic options for the 2019 novel coronavirus (2019-nCoV). Nat Rev Drug Discov 2020.
37. Li W, Moore MJ, Vasilieva N, et al. Angiotensin-converting enzyme 2 is a functional receptor for the SARS coronavirus. Nature 2003;426:450-4. doi: 10.1038/nature02145.
38. Pan H, Peto R, Henao-Restrepo AM, et al. Repurposed Antiviral Drugs for Covid-19 - Interim WHO Solidarity Trial Results. N Engl J Med 2020.
39. Wu CY, Jan JT, Ma SH, et al. Small molecules targeting severe acute respiratory syndrome human coronavirus. Proc Natl Acad Sci U S A 2004;101:10012-7. doi: 10.1073/pnas.0403596101. Epub 2004 Jun 28.
40. de Wilde AH, Jochmans D, Posthuma CC, et al. Screening of an FDA-approved compound library identifies four small-molecule inhibitors of Middle East respiratory syndrome coronavirus replication in cell culture. Antimicrob Agents Chemother 2014;58:4875-84. doi: 10.1128/AAC.03011-14. Epub 2014 May 19.
41. Chan JF, Yao Y, Yeung ML, et al. Treatment With Lopinavir/Ritonavir or Interferon-beta1b Improves Outcome of MERS-CoV Infection in a Nonhuman Primate Model of Common Marmoset. J Infect Dis 2015;212:1904-13. doi: 10.093/infdis/jiv392. Epub 2015 Jul 21.
42. Chinese Clinical Trial Registry. http://www.chictr.org.cn/enindex.aspx.
43. Cao B, Wang Y, Wen D, et al. A Trial of Lopinavir-Ritonavir in Adults Hospitalized with Severe Covid-19. N Engl J Med 2020;382:1787-99.
44. Cao B, Wang Y, Wen D, et al. A Trial of Lopinavir-Ritonavir in Adults Hospitalized with Severe Covid-19. N Engl J Med 2020.
45. Dalerba P, Levin B, Thompson JL. A Trial of Lopinavir-Ritonavir in Covid-19. N Engl J Med 2020;382.
46. Kunz KM. A Trial of Lopinavir-Ritonavir in Covid-19. N Engl J Med 2020;382.
47. Corrao S, Natoli G, Cacopardo B. A Trial of Lopinavir-Ritonavir in Covid-19. N Engl J Med 2020;382.
48. Carmona-Bayonas A, Jimenez-Fonseca P, Castanon E. A Trial of Lopinavir-Ritonavir in Covid-19. N Engl J Med 2020;382.
49. Havlichek D, Jr. A Trial of Lopinavir-Ritonavir in Covid-19. N Engl J Med 2020;382.
50. Lopinavir-ritonavir in patients admitted to hospital with COVID-19 (RECOVERY): a randomised, controlled, open-label, platform trial. Lancet 2020;396:1345-52.
51. Abbvie Corporation. Product Monograph: Kaletra® lopinavir/ritonavir film-coated tablets (100/25 mg, 200/50 mg). 2019.

52. Murphy RL, Brun S, Hicks C, et al. ABT-378/ritonavir plus stavudine and lamivudine for the treatment of antiretroviral-naive adults with HIV-1 infection: 48-week results. AIDS 2001;15:F1-9. doi: 10.1097/00002030-200101050-00002.
53. Walmsley S, Bernstein B, King M, et al. Lopinavir-ritonavir versus nelfinavir for the initial treatment of HIV infection. N Engl J Med 2002;346:2039-46. doi: 10.1056/NEJMoa012354.
54. Johnson MA, Gathe JC, Jr., Podzamczer D, et al. A once-daily lopinavir/ritonavir-based regimen provides noninferior antiviral activity compared with a twice-daily regimen. J Acquir Immune Defic Syndr 2006;43:153-60. doi: 10.1097/01.qai.0000242449.67155.1a.
55. Gathe J, da Silva BA, Cohen DE, et al. A once-daily lopinavir/ritonavir-based regimen is noninferior to twice-daily dosing and results in similar safety and tolerability in antiretroviral-naive subjects through 48 weeks. J Acquir Immune Defic Syndr 2009;50:474-81. doi: 10.1097/QAI.0b013e31819c2937.
56. Leal L, Leon A, Torres B, et al. A randomized clinical trial comparing ritonavir-boosted lopinavir versus maraviroc each with tenofovir plus emtricitabine for post-exposure prophylaxis for HIV infection. J Antimicrob Chemother 2016;71:1982-6. doi: 10.093/jac/dkw048. Epub 2016 Mar 18.
57. Leal L, Leon A, Torres B, et al. A randomized clinical trial comparing ritonavir-boosted lopinavir versus raltegravir each with tenofovir plus emtricitabine for post-exposure prophylaxis for HIV infection. J Antimicrob Chemother 2016;71:1987-93. doi: 10.093/jac/dkw049. Epub 2016 Mar 18.
58. Fatkenheuer G, Jessen H, Stoehr A, et al. PEPDar: A randomized prospective noninferiority study of ritonavir-boosted darunavir for HIV post-exposure prophylaxis. HIV Med 2016;17:453-9. doi: 10.1111/hiv.12363. Epub 2016 May 11.
59. Goodman RA, Orenstein WA, Munro TF, Smith SC, Sikes RK. Impact of influenza A in a nursing home. JAMA 1982;247:1451-3.
60. Guan W-J, Ni Z-Y, Hu Y, et al. Clinical characteristics of 2019 novel coronavirus infection in China. medRxiv 2020.
61. Ofner M, Lem M, Sarwal S, Vearncombe M, Simor A. Cluster of severe acute respiratory syndrome cases among protected health care workers-Toronto, April 2003. Can Commun Dis Rep 2003;29:93-7.
62. Campbell JA. The SARS Commission: Commission to Investigate the Introduction and Spread of SARS in Ontario 2006.
63. Arabi YM, Balkhy HH, Hayden FG, et al. Middle East Respiratory Syndrome. N Engl J Med 2017;376:584-94. doi: 10.1056/NEJMsr1408795.
64. Jiang L, McGeer A, McNeil S, et al. Which healthcare workers work with acute respiratory illness? Evidence from Canadian acute-care hospitals during 4 influenza seasons: 2010-2011 to 2013-2014. Infect Control Hosp Epidemiol 2019;40:889-96. doi: 10.1017/ice.2019.141. Epub Jun 18.
65. Kang L, Li Y, Hu S, et al. The mental health of medical workers in Wuhan, China dealing with the 2019 novel coronavirus. Lancet Psychiatry 2020;5:30047-X.
66. Public Health Agency of Canada. Public health management of cases and contacts associated with novel coronavirus disease 2019 (COVID-19). Available at: https://www.canada.ca/en/public-health/services/diseases/2019-novel-coronavirus-

infection/health-professionals/interim-guidance-cases-contacts.html#co. Accessed March 18, 2020.
67. Henao-Restrepo AM, Camacho A, Longini IM, et al. Efficacy and effectiveness of an rVSV-vectored vaccine in preventing Ebola virus disease: final results from the Guinea ring vaccination, open-label, cluster-randomised trial (Ebola Ca Suffit!). Lancet 2017;389:505-18. doi: 10.1016/S0140-6736(16)32621-6. Epub 2016 Dec 23.
68. Henao-Restrepo AM, Longini IM, Egger M, et al. Efficacy and effectiveness of an rVSV-vectored vaccine expressing Ebola surface glycoprotein: interim results from the Guinea ring vaccination cluster-randomised trial. Lancet 2015;386:857-66. doi: 10.1016/S0140-6736(15)61117-5. Epub 2015 Aug 3.
69. The ring vaccination trial: a novel cluster randomised controlled trial design to evaluate vaccine efficacy and effectiveness during outbreaks, with special reference to Ebola. BMJ 2015;351:h3740.:10.1136/bmj.h3740.
70. Kretzschmar M, van den Hof S, Wallinga J, van Wijngaarden J. Ring vaccination and smallpox control. Emerg Infect Dis 2004;10:832-41. doi: 10.3201/eid1005.030419.
71. Riley S, Fraser C, Donnelly CA, et al. Transmission dynamics of the etiological agent of SARS in Hong Kong: impact of public health interventions. Science 2003;300:1961-6. doi: 10.126/science.1086478. Epub 2003 May 23.
72. Brown CH, Ten Have TR, Jo B, et al. Adaptive designs for randomized trials in public health. Annu Rev Public Health 2009;30:1-25.:10.1146/annurev.publhealth.031308.100223.
73. Thorlund K, Haggstrom J, Park JJ, Mills EJ. Key design considerations for adaptive clinical trials: a primer for clinicians. BMJ 2018;360:k698.:10.1136/bmj.k698.
74. Peto R, Pike MC, Armitage P, et al. Design and analysis of randomized clinical trials requiring prolonged observation of each patient. I. Introduction and design. Br J Cancer 1976;34:585-612. doi: 10.1038/bjc.976.220.
75. Division of AIDS (DAIDS) Table for Grading the Severity of Adult and Pediatric Adverse Events, Version 2.0, November 2014. Division of AIDS, National Institute of Allergy and Infectious Diseases, National Institutes of Health, US Department of Health and Human Services; 2014.
76. Furukawa TA, Kessler RC, Slade T, Andrews G. The performance of the K6 and K10 screening scales for psychological distress in the Australian National Survey of Mental Health and Well-Being. Psychol Med 2003;33:357-62. doi: 10.1017/s0033291702006700.
77. Kessler RC, Andrews G, Colpe LJ, et al. Short screening scales to monitor population prevalences and trends in non-specific psychological distress. Psychol Med 2002;32:959-76. doi: 10.1017/s0033291702006074.
78. Horowitz M, Wilner N, Alvarez W. Impact of Event Scale: a measure of subjective stress. Psychosom Med 1979;41:209-18. doi: 10.1097/00006842-197905000-00004.
79. Maunder RG, Lancee WJ, Balderson KE, et al. Long-term psychological and occupational effects of providing hospital healthcare during SARS outbreak. Emerg Infect Dis 2006;12:1924-32. doi: 10.3201/eid1212.060584.
80. https://euroqol.org/eq-5d-instruments/eq-5d-5l-about/.
81. Guidelines for the economic evaluation of health technologies: Canada. 4th ed. Ottawa: CADTH; 2017 Mar.

82. Drummond MF, Sculpher MJ, Claxton K, Stoddart GL, Torrance GW. Methods for the economic evaluation of health care programmes (fourth edition). Oxford University Press. 2015.
83. Sullivan SD, Mauskopf JA, Augustovski F, et al. Budget impact analysis-principles of good practice: report of the ISPOR 2012 Budget Impact Analysis Good Practice II Task Force. Value Health 2014;17:5-14. doi: 0.1016/j.jval.2013.08.291. Epub Dec 13.
84. Canadian Institutes of Health Research, Natural Sciences and Engineering Research Council of Canada, and Social Sciences and Humanities Research Council, Tri-Council Policy Statement: Ethical Conduct for Research Involving Humans, December 2018.
85. Kumar GN, Dykstra J, Roberts EM, et al. Potent inhibition of the cytochrome P-450 3A-mediated human liver microsomal metabolism of a novel HIV protease inhibitor by ritonavir: A positive drug-drug interaction. Drug Metab Dispos 1999;27:902-8.
86. Backer JA, Klinkenberg D, Wallinga J. Incubation period of 2019 novel coronavirus (2019-nCoV) infections among travellers from Wuhan, China, 20-28 January 2020. Euro Surveill 2020;25(5).10.2807/1560-7917.ES.2020.25.5.2000062.
87. Assiri A, McGeer A, Perl TM, et al. Hospital outbreak of Middle East respiratory syndrome coronavirus. N Engl J Med 2013;369:407-16. doi: 10.1056/NEJMoa1306742. Epub 2013 Jun 19.
88. Jiang X, Rayner S, Luo MH. Does SARS-CoV-2 has a longer incubation period than SARS and MERS? J Med Virol 2020;13:25708.
89. Lauer SA, Grantz KH, Bi Q, et al. The Incubation Period of Coronavirus Disease 2019 (COVID-19) From Publicly Reported Confirmed Cases: Estimation and Application. Ann Intern Med 2020;10:M20-0504.
90. Molina JM, Podsadecki TJ, Johnson MA, et al. A lopinavir/ritonavir-based once-daily regimen results in better compliance and is non-inferior to a twice-daily regimen through 96 weeks. AIDS Res Hum Retroviruses 2007;23:1505-14. doi: 10.089/aid.2007.0107.
91. Lafeuillade A, Dupon M, Livrozet JM, Morand-Joubert L, Cohen Codar I, Allaert FA. Efficacy and Tolerance of Lopinavir/ritonavir in Clinical Practice: An Observational Prospective Cohort of 1278 Patients. Proceedings of the 7th International Congress on Drug Therapy in HIV Infection Poster 140. Glasgow, UK2004.
92. Personal communication, Rick Galli, Chief Technical Officer, bioLytical Laboratories, March 16, 2020.
93. Larios OE, Coleman BL, Drews SJ, et al. Self-collected mid-turbinate swabs for the detection of respiratory viruses in adults with acute respiratory illnesses. PLoS One 2011;6:e21335. doi: 10.1371/journal.pone.0021335. Epub 2011 Jun 23.
94. Granados A, Quach S, McGeer A, Gubbay JB, Kwong JC. Detecting and quantifying influenza virus with self- versus investigator-collected mid-turbinate nasal swabs. J Med Virol 2017;89:1295-9. doi: 10.002/jmv.24753. Epub 2017 Feb 27.
95. Corman VM, Landt O, Kaiser M, et al. Detection of 2019 novel coronavirus (2019-nCoV) by real-time RT-PCR. Euro Surveill 2020;25(3).10.2807/1560-7917.ES.2020.25.3.2000045.

96. Ali M, Han S, Gunst CJ, Lim S, Luinstra K, Smieja M. Throat and nasal swabs for molecular detection of respiratory viruses in acute pharyngitis. Virol J 2015;12:178.:10.1186/s12985-015-0408-z.
97. Van Wesenbeeck L, Meeuws H, D'Haese D, et al. Sampling variability between two mid-turbinate swabs of the same patient has implications for influenza viral load monitoring. Virol J 2014;11:233.:10.1186/s12985-014-0233-9.
98. We ask because we care: The Tri-Hospital + TPH Health Equity Data Collection Research Project Report. Toronto 2013.
99. Buckrell S, Coleman BL, McNeil SA, et al. Sources of viral respiratory infections in Canadian acute care hospital healthcare personnel. J Hosp Infect 2020;16:30012-8.
100. Matsuishi K, Kawazoe A, Imai H, et al. Psychological impact of the pandemic (H1N1) 2009 on general hospital workers in Kobe. Psychiatry Clin Neurosci 2012;66:353-60. doi: 10.1111/j.440-819.2012.02336.x.
101. Wang Y, Xu B, Zhao G, Cao R, He X, Fu S. Is quarantine related to immediate negative psychological consequences during the 2009 H1N1 epidemic? Gen Hosp Psychiatry 2011;33:75-7. doi: 10.1016/j.genhosppsych.2010.11.001. Epub Dec 13.
102. Hussain H, McGeer A, McNeil S, et al. Factors associated with influenza vaccination among healthcare workers in acute care hospitals in Canada. Influenza Other Respir Viruses 2018;12:319-25. doi: 10.1111/irv.12545. Epub 2018 Mar 2.
103. Duthaler U, Berger B, Erb S, et al. Using dried blood spots to facilitate therapeutic drug monitoring of antiretroviral drugs in resource-poor regions. J Antimicrob Chemother 2018;73:2729-37. doi: 10.1093/jac/dky254.
104. Kromdijk W, Mulder JW, Smit PM, Ter Heine R, Beijnen JH, Huitema AD. Therapeutic drug monitoring of antiretroviral drugs at home using dried blood spots: a proof-of-concept study. Antivir Ther 2013;18:821-5. doi: 10.3851/IMP2501. Epub 012 Dec 12.
105. Ghosn J, Flandre P, Cohen-Codar I, et al. Long-term (96-week) follow-up of antiretroviral-naive HIV-infected patients treated with first-line lopinavir/ritonavir monotherapy in the MONARK trial. HIV Med 2010;11:137-42. doi: 10.1111/j.468-293.2009.00752.x. Epub 2009 Aug 13.
106. Delfraissy JF, Flandre P, Delaugerre C, et al. Lopinavir/ritonavir monotherapy or plus zidovudine and lamivudine in antiretroviral-naive HIV-infected patients. AIDS 2008;22:385-93. doi: 10.1097/QAD.0b013e3282f3f16d.
107. Pang X, Zhu Z, Xu F, et al. Evaluation of control measures implemented in the severe acute respiratory syndrome outbreak in Beijing, 2003. JAMA 2003;290:3215-21. doi: 10.1001/jama.290.24.3215.
108. Goh DL, Lee BW, Chia KS, et al. Secondary household transmission of SARS, Singapore. Emerg Infect Dis 2004;10:232-4. doi: 10.3201/eid1002.030676.
109. Personal communication: McGeer, A., February 14, 2020.
110. Muller MP, Richardson SE, McGeer A, et al. Early diagnosis of SARS: lessons from the Toronto SARS outbreak. Eur J Clin Microbiol Infect Dis 2006;25:230-7. doi: 10.1007/s10096-006-0127-x.
111. Thai PQ, Mai le Q, Welkers MR, et al. Pandemic H1N1 virus transmission and shedding dynamics in index case households of a prospective Vietnamese cohort. J Infect 2014;68:581-90. doi: 10.1016/j.jinf.2014.01.008. Epub Feb 1.

112. Petrie JG, Ohmit SE, Cowling BJ, et al. Influenza transmission in a cohort of households with children: 2010-2011. PLoS One 2013;8:e75339. doi: 10.1371/journal.pone.0075339. eCollection 2013.
113. Carcione D, Giele CM, Goggin LS, et al. Secondary attack rate of pandemic influenza A(H1N1) 2009 in Western Australian households, 29 May-7 August 2009. Euro Surveill 2011;16:3.
114. Cauchemez S, Donnelly CA, Reed C, et al. Household transmission of 2009 pandemic influenza A (H1N1) virus in the United States. N Engl J Med 2009;361:2619-27. doi: 10.1056/NEJMoa0905498.
115. Kwok KO, Tang A, Wei VWI, Park WH, Yeoh EK, Riley S. Epidemic Models of Contact Tracing: Systematic Review of Transmission Studies of Severe Acute Respiratory Syndrome and Middle East Respiratory Syndrome. Comput Struct Biotechnol J 2019;17:186-194.:10.1016/j.csbj.2019.01.003. eCollection .
116. Hellewell J, Abbott S, Gimma A, et al. Feasibility of controlling 2019-nCoV outbreaks by isolation of cases and contacts. medRxiv 2020:2020.02.08.20021162.
117. Donner A, Klar N. Pitfalls of and controversies in cluster randomization trials. Am J Public Health 2004;94:416-22. doi: 10.2105/ajph.94.3.416.
118. Lake S, Kammann E, Klar N, Betensky R. Sample size re-estimation in cluster randomization trials. Stat Med 2002;21:1337-50. doi: 10.002/sim.121.
119. O'Brien PC, Fleming TR. A multiple testing procedure for clinical trials. Biometrics 1979;35:549-56.
